# MicroRNAs in veterinary viral diseases: A comprehensive review from molecular mechanisms to clinical translation

**DOI:** 10.1016/j.virusres.2026.199800

**Published:** 2026-09-16

**Authors:** Mohamad Reza Choobineh, Daryoosh Choobineh, Behzad Hajieghrari

**Affiliations:** a Department of Clinical Sciences, Faculty of Veterinary Medicine, Kaz. C. Islamic Azad University, Kazerun, Iran; b University of Applied Science and Technology, Fars, Iran; c Department of Agricultural Biotechnology, College of Agriculture, Jahrom University, Jahrom, P.O. Box 74135-111, Iran

**Keywords:** MicroRNAs (miRNAs), Veterinary viral diseases, Virus–host interactions, Pathogenesis, Diagnostic biomarkers, Antiviral therapy, One Health

## Abstract

MicroRNAs (miRNAs) are small non-coding RNA molecules, approximately 22 nucleotides in length, that regulate post-transcriptional gene expression and have emerged as pivotal modulators of host–virus interactions. Veterinary viral diseases continue to pose substantial challenges to animal health, livestock productivity, food security, and public health, particularly due to their zoonotic potential. While miRNA research has advanced considerably, a comprehensive and critically integrated understanding of their biological functions and clinical applications across veterinary viral diseases remains incomplete. This comprehensive critical narrative synthesis addresses four overarching research questions: (1) What conserved and species-specific miRNA-mediated mechanisms govern major veterinary viral diseases? (2) What contextual factors determine antiviral vs. proviral duality? (3) To what extent do circulating miRNA signatures offer diagnostic and prognostic utility? (4) What translational barriers currently prevent clinical implementation, and how can the One Health framework help overcome them? Integrating three interconnected dimensions—molecular mechanisms, pathogen-specific responses, and translational applications—the review synthesizes evidence across PRRSV, avian oncogenic viruses (MDV, ALV), the immunosuppressive IBDV, FMD, BVDV, Ebola, Hendra, Rabies, and aquatic viral diseases. A key contribution of this review is the proposal of a four-axis contextual framework that explains the antiviral/proviral duality of miRNAs, and a 'One miRNA, One Health' convergence model with a concrete implementation roadmap. Key findings include: (a) a four-axis contextual framework (cell type, infection stage, viral strain, host–viral miRNA competition) that explains the antiviral/proviral duality; (b) virus-encoded miRNAs (v-miRNAs) as lower-risk therapeutic targets due to their absence from uninfected host genomes; (c) circulating miRNA biomarkers validated only at proof-of-concept stage (TRL 1–3), with no veterinary product yet at TRL ≥4; and (d) zoonotic conservation of miR-155, miR-146a, miR-21, and miR-122 across human and veterinary pathogens, supporting a ‘One miRNA, One Health’ convergence strategy. Critical short-term priorities are standardized pre-analytical protocols, open-access veterinary miRNA databases, and multicenter validation in natural infection cohorts.

## Highlights

• MicroRNAs (miRNAs) are key regulators of host–virus interactions in veterinary viral diseases.
• A four-axis contextual framework explains antiviral versus proviral duality of miRNAs.
• Virus-encoded miRNAs (v-miRNAs) represent promising lower-risk therapeutic targets.
• No veterinary miRNA-based product has yet reached Technology Readiness Level (TRL) ≥4.
• Conserved miRNAs (miR-155, miR-146a, miR-21, miR-122) support a "One miRNA, One Health" strategy.

## Graphical abstract

From Molecular Mechanisms to One Health Translation in Veterinary Viral Diseases. It summarizes the tripartite conceptual framework of the review. The left panel illustrates the canonical and non-canonical miRNA biogenesis pathways leading to mature miRNA-loaded RISC complexes. The central panel depicts the context-dependent duality of miRNA functions (antiviral versus proviral) across major veterinary pathogens, governed by the four-axis framework (cell type, infection stage, viral strain, and host–viral miRNA competition). The right panel highlights translational applications including circulating miRNA biomarkers, miRNA-based therapeutics, and vaccine adjuvants, all integrated under the "One miRNA, One Health" convergence strategy. Current progress remains at the proof-of-concept stage (TRL 1–3).

**Figure:**
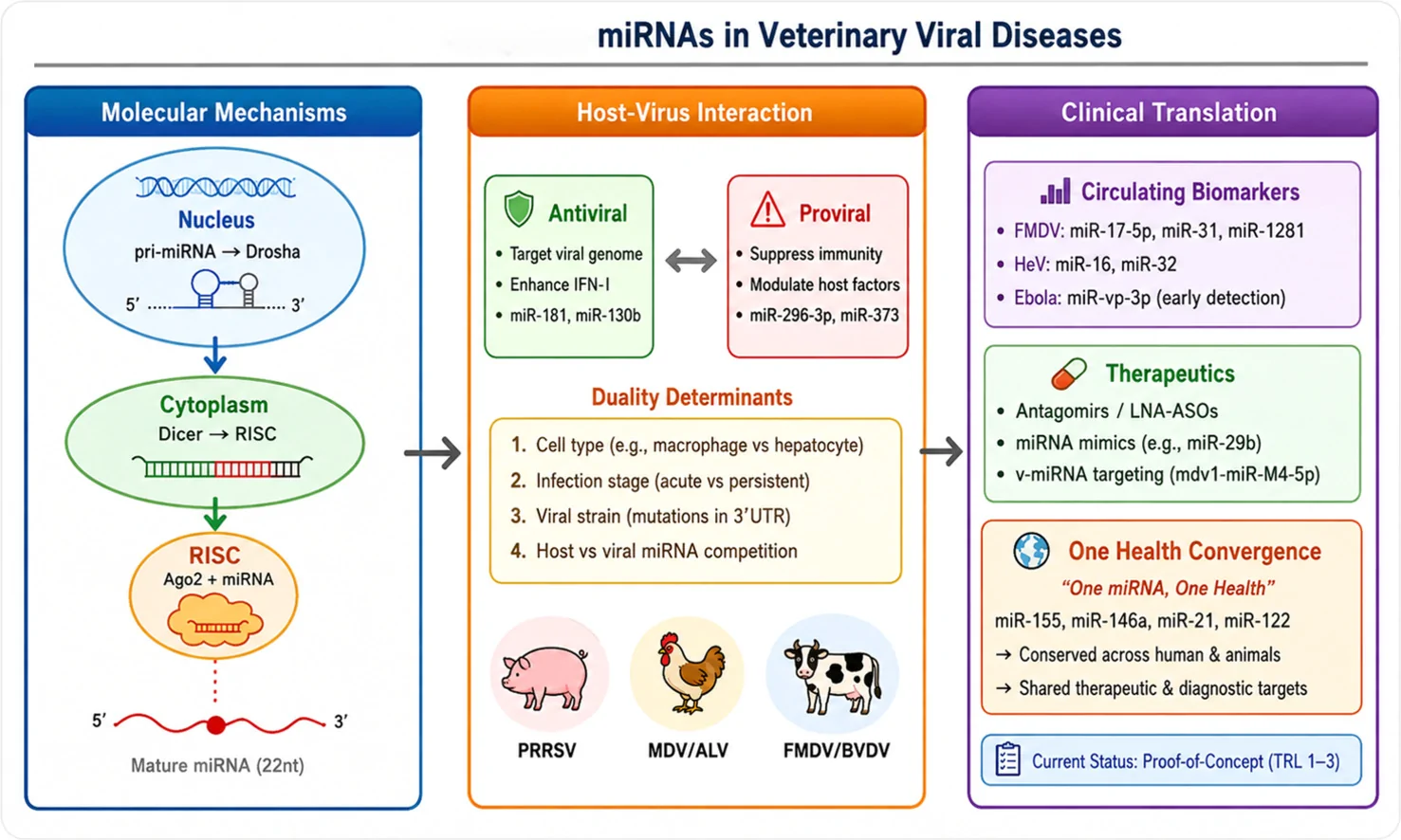

## 1 Introduction

Viral infections remain among the leading causes of morbidity and mortality in animal populations worldwide (Yadav et al., 2019), imposing substantial economic burdens through reduced livestock productivity, increased production costs, trade restrictions, and threats to global food security. The economic impact of viral diseases in production animals is staggering: porcine reproductive and respiratory syndrome virus (PRRSV) alone causes an estimated annual loss of nearly $600 million in the United States (Zhang et al., 2026). Marek's disease in poultry has been associated with annual economic losses exceeding USD 1–2 billion worldwide, due to mortality, condemnations, reduced growth performance, and costs associated with vaccination and control measures (Fotouh et al., 2026). Beyond their impact on animal health, many veterinary viral pathogens possess zoonotic potential, enabling transmission between animals and humans and posing significant public health concerns (Rahman et al., 2020). The emergence and re-emergence of viral diseases has been accelerated by factors such as climate change, globalization of animal trade, increased animal movement, urbanization, and changes in vector ecology (Rahman et al., 2020). Consequently, the development of innovative diagnostic, preventive, and therapeutic approaches has become a major priority in veterinary medicine.

Advances in molecular biology, particularly in the last two decades, have considerably expanded our understanding of viral pathogenesis and host–pathogen interactions (Strauss and Strauss, 2008; Bartel, 2004). Among the diverse classes of regulatory molecules, microRNAs (miRNAs) have emerged as key post-transcriptional regulators, owing to their ability to modulate gene expression, target specific messenger RNAs, and remain readily detectable in a wide range of biological fluids (Jorge et al., 2021). Unlike messenger RNAs, miRNAs are remarkably stable in formalin-fixed, paraffin-embedded tissues and in archived biological samples, making them attractive candidates for molecular biomarkers (Hall et al., 2012). Furthermore, miRNAs exhibit cross-species conservation—many miRNAs are evolutionarily conserved across vertebrates, with identities often exceeding 95% between species (Samir and Pessler, 2016). For example, miR-155-5p, miR-18a-5p, and miR-34a-5p show 100% identity between humans and chickens, while miR-29a shows 95.2% identity, suggesting that the majority of miRNAs constitute a large class of predominantly orthologous or homologous molecules (Samir and Pessler, 2016). This facilitates translational research across veterinary species.

The biogenesis of miRNAs is a tightly regulated and highly coordinated process that underpins their biological activity (Ha and Kim, 2014; MacFarlane and Murphy, 2010). Through precise temporal and spatial regulation, miRNAs contribute to essential cellular processes, including growth, differentiation, apoptosis, and maintenance of cellular homeostasis (Ha and Kim, 2014; Ghai and Wang, 2016). Increasing evidence from both in vitro and in vivo studies has demonstrated that viral infections can profoundly alter host miRNA expression profiles (Drury et al., 2017). These alterations influence the expression of numerous genes involved in immune responses, inflammation, cell-cycle regulation, and viral replication, thereby shaping both viral pathogenesis and host defense mechanisms (Croce, 2009; Barbu et al., 2020).

Despite substantial advances, a comprehensive understanding of the biological and clinical significance of miRNAs across veterinary viral diseases remains incomplete. Although numerous studies have demonstrated the regulatory roles of miRNAs in host–virus interactions, current knowledge remains fragmented across different animal species and viral infections (Dong et al., 2017).

### 1.1 Aims and conceptual framework

Therefore, this narrative review aims to address the following overarching research questions: (1) What are the conserved and species-specific miRNA-mediated regulatory mechanisms across major veterinary viral diseases? (2) How do host- and virus-encoded miRNAs orchestrate the dual (antiviral/proviral) functions during infection, and what determines this contextual duality? (3) To what extent do circulating miRNA signatures demonstrate diagnostic and prognostic utility across different veterinary viral pathogens? (4) What are the critical translational barriers that currently prevent routine clinical implementation of miRNA-based therapies in veterinary practice, and how can they be overcome within the One Health framework?

To answer these questions, we propose a conceptual framework that integrates three interconnected dimensions: (a) **molecular mechanisms** — examining miRNA biogenesis, target recognition, and regulatory networks; (b) **pathogen-specific responses** — comparing miRNA signatures across viral families and host species; and (c) **translational applications** — critically evaluating biomarkers, therapeutics, and vaccine adjuvants. This tripartite framework guides the systematic synthesis of evidence presented herein and identifies knowledge gaps that must be prioritized for future research. By moving beyond descriptive cataloguing towards mechanistic interpretation and cross-disease comparative analysis, this review seeks to provide actionable insights for both basic researchers and clinical veterinarians.

## 2 Literature search strategy

This narrative review was conducted to provide a comprehensive and critically integrated overview of current knowledge on the roles of microRNAs in veterinary viral infections. A systematic literature search was performed using the electronic databases PubMed, Scopus, Web of Science, and Google Scholar to identify relevant peer-reviewed articles published in English between 2000 and 2026. The search strategy incorporated combinations of Medical Subject Headings (MeSH) and free-text keywords, including: microRNA, miRNA, veterinary viral infections, animal viruses, circulating microRNAs, biomarkers, RNA therapeutics, antiviral therapy, livestock, together with the names of major veterinary viral diseases (e.g., PRRSV, FMDV, BVDV, MDV, ALV, IBDV, VHSV, HeV). Specialist databases miRBase (v22) and Gene Expression Omnibus (GEO) were also searched for publicly deposited veterinary miRNA expression datasets. Inclusion criteria required: (1) original research or systematic reviews providing experimental evidence on miRNA functions in veterinary viral infections; (2) studies reporting quantitative miRNA expression data, validated target interactions (luciferase reporter assay, qRT-PCR, or Western blot), or therapeutic applications; and (3) studies with clear methodological descriptions. Exclusion criteria were applied as described in Section 2 (search date: January 2026). For study quality, the following signals were assessed: presence of an appropriate uninfected control group; sample size ≥5 biological replicates per group for in vitro studies or ≥3 animals per group for in vivo studies; and use of at least one orthogonal validation method beyond discovery-phase sequencing. Studies providing only bioinformatic predictions without experimental validation of at least one miRNA–target pair were included where directly relevant but are flagged as lower-confidence in the text. No formal risk-of-bias tool was applied given the narrative scope; quality signals are reported descriptively. Additional references were identified through manual screening of reference lists of selected publications. The collected literature was critically reviewed and synthesized to present an integrated perspective on the current understanding of miRNA biology in veterinary virology, while highlighting recent advances, existing knowledge gaps, and future research directions.

## 3 MicroRNA biogenesis: canonical and non-canonical pathways

MicroRNAs are a class of small, single-stranded, non-coding RNA molecules approximately 22 nucleotides in length (Ha and Kim, 2014; Farrokhi and Hajieghrari, 2020). Most miRNAs are initially transcribed from the genome as primary miRNA transcripts (pri-miRNAs), predominantly by RNA polymerase II and, less frequently, by RNA polymerase III (Borchert et al., 2006). These primary transcripts undergo a series of processing steps to generate mature miRNAs (Fig. 1). Approximately 50% of identified miRNAs are intragenic, originating primarily from intronic regions and, to a lesser extent, from exonic regions of protein-coding genes (Kim and Kim, 2007; de Rie et al., 2017). The remaining miRNAs are intergenic and are regulated by independent promoters (de Rie et al., 2017). Although miRNA genes are distributed throughout the genome (Lim et al., 2003a; Lim et al., 2003b), some functionally related miRNAs are organized into genomic clusters, commonly transcribed as polycistronic transcripts and often sharing similar seed sequences, thereby forming members of the same miRNA family (Lagos-Quintana et al., 2001; Ambros, 2004).

**Fig. 1:**
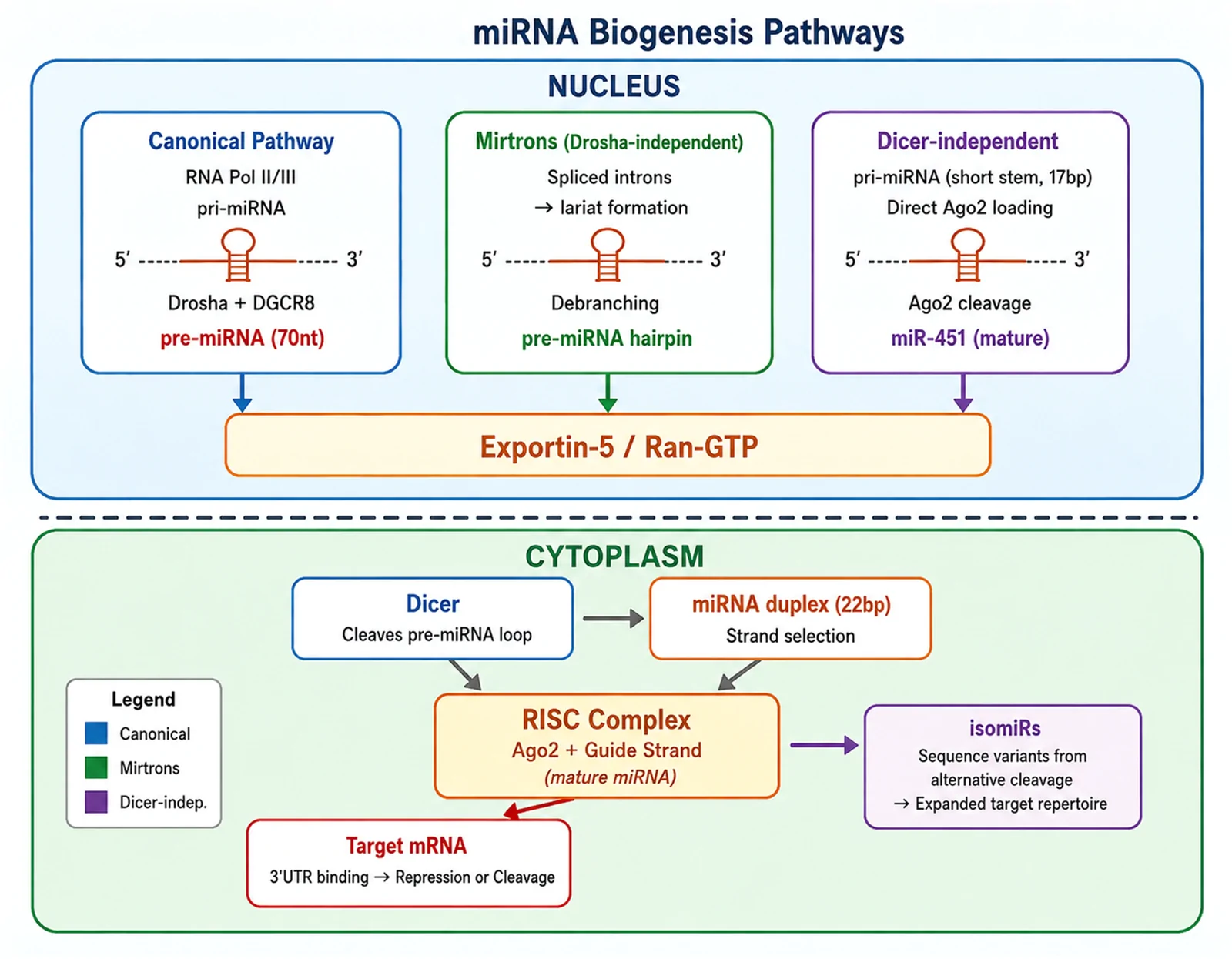
Canonical and non-canonical pathways of MicroRNA biogenesis. Schematic representation of miRNA biogenesis in eukaryotic cells. In the nucleus, RNA polymerase II/III transcribes primary miRNAs (pri-miRNAs) that are processed by the Drosha–DGCR8 microprocessor complex into precursor miRNAs (pre-miRNAs, ∼70 nt). Alternatively, mirtrons originate from spliced introns and bypass Drosha processing. Non-canonical Dicer-independent miRNAs (e.g., miR-451) are loaded directly onto Argonaute 2 (Ago2). All pre-miRNAs are exported to the cytoplasm via Exportin-5/Ran-GTP. In the cytoplasm, Dicer cleaves the pre-miRNA terminal loop to generate a ∼22 bp duplex. Strand selection incorporates the guide strand into the RNA-induced silencing complex (RISC) with Ago2, forming the mature miRISC that targets complementary mRNAs (predominantly in the 3′UTR), leading to translational repression or mRNA cleavage. IsomiRs, arising from alternative cleavage or 3′ end modifications, expand the regulatory repertoire. Veterinary relevance: the high cross-species conservation of miRNA sequences (e.g., miR-155, miR-146a) facilitates translational research across livestock, poultry, and companion animals.

### 3.1 Canonical miRNA biogenesis pathway

In the canonical pathway, pri-miRNAs transcribed by RNA polymerase II within the nucleus subsequently fold into one or more characteristic hairpin structures (Lee et al., 2003; Ho et al., 2022). Each hairpin typically contains an imperfect stem-loop structure with a stem of approximately 30–35 base pairs and a terminal loop (Cullen, 2004). These structures are recognized and processed by the Microprocessor complex, composed of the RNase III endonuclease Drosha and its cofactor DGCR8, generating a precursor miRNA (pre-miRNA) of approximately 70–100 nucleotides in length (Denli et al., 2004; O'Brien et al., 2018). The pre-miRNA is subsequently exported from the nucleus to the cytoplasm via nuclear pore complexes by Exportin-5 (XPO5) in a Ran-GTP-dependent manner (Yi et al., 2003).

In the cytoplasm, the RNase III enzyme Dicer cleaves the terminal loop of the pre-miRNA, producing a double-stranded miRNA duplex (Bartel, 2004; Zhang et al., 2004). The resulting miRNA duplex is then unwound by helicase-associated factors, producing two single-stranded RNA molecules. One strand is selectively retained as the guide strand, which represents the mature and biologically active miRNA. The complementary passenger strand is typically excluded from the silencing machinery and degraded (Medley et al., 2021). The guide strand is then loaded onto an Argonaute (Ago) protein, a key component of the RNA-induced silencing complex (RISC), forming the functional miRISC complex (Leitão and Enguita, 2022). Depending on the specific miRNA, the RISC complex predominantly binds to complementary sequences within the 3′ untranslated region (3′UTR) of target messenger RNAs, though interactions with the 5′ untranslated region (5′UTR), coding sequences, and promoter regions have also been reported (Xu et al., 2014; O'Brien et al., 2018).

### 3.2 Non-canonical miRNA biogenesis pathways

Several non-canonical miRNA biogenesis pathways have been described, utilizing alternative combinations of processing factors, including Drosha, Dicer, Exportin-5, and AGO2 (O'Brien et al., 2018; Samir and Pessler, 2016). These pathways are broadly categorized into Drosha/DGCR8-independent and Dicer-independent mechanisms (O'Brien et al., 2018; Samir and Vaas, 2016).

**Mirtrons** constitute a well-characterized class of miRNAs produced through a Drosha-independent pathway and are derived from spliced introns processed by the spliceosomal machinery (Babiarz et al., 2008; O'Brien et al., 2018). Following splicing and debranching, the intronic lariat forms a pre-miRNA hairpin that bypasses Drosha processing and enters the canonical pathway at the Exportin-5-mediated nuclear export step (O'Brien et al., 2018).

**Dicer-independent miRNAs**, exemplified by miR-451, undergo maturation independently of Dicer but require Argonaute proteins (Cheloufi et al., 2010). This distinctive processing mechanism is largely attributed to the unusually short stem length of their pre-miRNA structures (approximately 17 base pairs), which prevents efficient recognition and cleavage by Dicer (Cheloufi et al., 2010). Instead, the pre-miRNA is directly loaded onto Ago2, which cleaves the hairpin, followed by 3′ resection via poly(A)-specific ribonuclease (PARN) to generate the mature miRNA (Cheloufi et al., 2010).

### 3.3 IsomiRs and miRNA diversity

Advances in high-throughput sequencing technologies, particularly next-generation sequencing (NGS), have revealed the existence of isomiRs—multiple sequence variants derived from a single canonical miRNA (Jafari and Sa, 2016; Lu et al., 2009). These variants arise from alternative Drosha and Dicer cleavage sites or through nucleotide additions at the 3′ end (Tomasello et al., 2021). IsomiRs represent distinct isoforms of conventional miRNAs with unique regulatory functions and target repertoires that differ from their corresponding reference miRNAs (Tomasello et al., 2021). miRNA isoforms exhibit considerable heterogeneity in sequence composition, length, or both, thereby contributing to the complexity of post-transcriptional gene regulation (Tomasello et al., 2021).

### 3.4 Implications for veterinary medicine

The complexity of miRNA biogenesis reflects the sophisticated regulatory networks that govern gene expression in eukaryotic cells. The coexistence of canonical and non-canonical biogenesis pathways, together with the generation of isomiRs, provides remarkable biological flexibility, enabling cells to adapt gene expression to diverse physiological and pathological conditions. Importantly, many miRNAs are evolutionarily conserved across species, with mature miRNA sequences often showing 95–100% identity between humans and chickens (Samir and Pessler, 2016). This cross-species conservation facilitates translational research across veterinary species. However, the generation of isomiRs, variations in processing efficiency, and species-specific differences in miRNA repertoires pose significant challenges to elucidating miRNA functions, accurately identifying target genes, and interpreting biological variability across tissues and species (Samir and Pessler, 2016; Samir and Vaas, 2016).

## 4 Biological functions and regulatory roles of microRNAs

### 4.1 Fundamental mechanisms of miRNA-mediated regulation

MicroRNAs are highly stable and remarkably efficient gene regulators that play pivotal roles across a wide range of physiological and pathological processes (Raisch et al., 2013). These small non-coding RNAs are involved in virtually every stage of biological development, including cellular growth, differentiation, proliferation, apoptosis, and tumorigenesis (Raisch et al., 2013; Shahini et al., 2018). The discovery of the first miRNA, lin-4, in the nematode Caenorhabditis elegans in 1993 marked a major breakthrough in molecular biology, revealing a novel layer of gene regulation beyond conventional transcriptional control (Shahini et al., 2018).

The regulatory network established between miRNAs and their target messenger RNAs (mRNAs) is highly complex and context-dependent, varying considerably among different cell types and tissues (Plotnikova et al., 2019; Grimson et al., 2007). The fate of a target mRNA is largely determined by the degree of sequence complementarity between the miRNA and its binding site. Perfect complementarity, a mechanism predominantly observed in plants, typically results in endonucleolytic cleavage of the target transcript (Skalsky and Cullen, 2010; Hajieghrari and Farrokhi, 2022). Conversely, the partial complementarity characteristic of mammalian and viral miRNAs initially induces translational repression, followed by reduced mRNA stability and eventual transcript degradation (Baek et al., 2008; Skalsky and Cullen, 2010).

### 4.2 Dual roles of host miRNAs in viral infection

A landmark study by Calin and colleagues provided the first direct evidence linking miRNA dysregulation to human disease by demonstrating the tumor-suppressive roles of miR-15 and miR-16 in chronic lymphocytic leukemia (Calin et al., 2002). Since then, miRNAs have been recognized as key regulators not only of host cellular pathways but also of host–pathogen interactions, particularly during viral infections (Asgari, 2011; Hajieghrari and Rahmanian Koshkaki, 2021).

Accumulating evidence indicates that host-derived miRNAs can exert either antiviral or proviral effects during viral infection (Zhang et al., 2026; Guo and Feng, 2025). In the context of PRRSV infection, research has identified 38 miRNAs to date, with 27 exhibiting antiviral properties and 11 promoting viral replication (Zhang et al., 2026; Guo and Feng, 2025). Antiviral miRNAs suppress viral infection through multiple mechanisms, including: direct targeting of viral genomes; modulation of entry receptor expression; regulation of critical host signaling pathways such as type I interferon (IFN-I) and NF-κB; and enhancement of innate immune responses (Zhang et al., 2026; Guo and Feng, 2025).

Conversely, proviral miRNAs facilitate viral replication by suppressing antiviral immune responses, modulating host factors essential for viral replication, or creating cellular environments permissive for viral propagation (Zhang et al., 2026; Guo and Feng, 2025).

Notably, several miRNAs—including miR-218, miR-122, miR-331-3p, miR-378, miR-24-3p, and miR-29a—have been reported to exert opposite effects on replication in different viral infection contexts, underscoring that miRNA-mediated regulation is highly context-dependent and influenced by cell type, viral strain, and infection stage (Guo and Feng, 2025).

### 4.3 Virus-encoded MicroRNAs (v-miRNAs)

Many viruses encode their own viral miRNAs (v-miRNAs), which regulate critical stages of the viral life cycle (Skalsky and Cullen, 2010; Gottwein and Cullen, 2008). To date, several avian viruses, mostly herpesviruses, have been shown to encode a number of novel miRNAs, including Marek's disease virus-1 (26 miRNAs), avirulent Marek's disease virus-2 (36 miRNAs), herpesvirus of turkeys (28 miRNAs), infectious laryngotracheitis virus (10 miRNAs), duck enteritis virus (33 miRNAs), and avian leukosis virus (2 miRNAs) (Fotouh et al., 2026). These molecules represent highly efficient regulatory elements that, unlike viral proteins, do not trigger host immune responses and occupy minimal genomic space (Skalsky and Cullen, 2010; Gottwein and Cullen, 2008).

Remarkably, MDV encodes a viral miRNA ortholog (mdv1-miR-M4-5p) that is indispensable for tumor induction in vivo, representing the first direct demonstration of a virus-encoded miRNA acting as a primary oncogenic factor in a natural host (Fotouh et al., 2026; Kappari et al., 2024; Bondada et al., 2019). This viral miRNA is a functional ortholog of gga-miR-155, and its critical role in oncogenicity has been firmly established (Fotouh et al., 2026; Bondada et al., 2019). In contrast, avian leukosis virus and reticuloendotheliosis virus exploit host miRNA pathways through insertional mutagenesis and NF-κB-mediated transcriptional activation, respectively, contributing to tumor progression in a context-dependent manner (Fotouh et al., 2026).

The multifunctional miR-155 pathway plays a central role in avian oncogenic virus-induced neoplastic diseases (Bondada et al., 2019). While oncogenic retroviruses such as ALV activate miR-155 by insertional activation, acutely transforming retroviruses use transduced oncogenes such as v-rel to upregulate miR-155 expression. MDV, on the other hand, encodes a functional miR-155 ortholog, demonstrating the oncogenic potential of the miR-155 pathway in cancers irrespective of the diverse etiology (Bondada et al., 2019).

From a therapeutic perspective, v-miRNAs represent particularly attractive drug targets compared with host miRNA-targeting strategies, for three principal reasons: (1) v-miRNAs are absent from the uninfected host genome, so inhibiting them would not disrupt normal host physiology and should reduce off-target toxicity; (2) the sequences of v-miRNAs are generally more conserved than those of rapidly mutating viral surface proteins, suggesting a higher barrier to resistance evolution under selective pressure from a small-RNA inhibitor; and (3) a single v-miRNA may simultaneously regulate dozens of host transcripts, meaning its inhibition can disrupt multiple immune-evasion mechanisms at once (Skalsky and Cullen, 2010). These properties have motivated proof-of-concept anti-v-miRNA studies, notably the targeting of mdv1-miR-M4-5p in MDV-infected cells, where inhibition reduces markers of cellular transformation (Kappari et al., 2024). Key outstanding challenges include efficient delivery of anti-v-miRNA agents to relevant cellular compartments in vivo, confirmatory in vivo efficacy data beyond cell lines, and systematic characterisation of the full targetome of each v-miRNA before clinical development is feasible.

### 4.4 Cross-species conservation of miRNAs

Most annotated miRNAs are evolutionarily conserved among a variety of organisms, particularly in their mature form, suggesting that the majority of miRNAs constitute a large class of predominantly orthologous or homologous molecules (Samir and Pessler, 2016). As exemplified by several miRNAs, cross-species variation in miRNA sequence is typically observed in 1 or 2 nucleotides in the periphery of the mature form and in its 3′ end, i.e., away from the highly conserved seed region (Samir and Pessler, 2016). This conservation has important implications for biomarker development and therapeutic applications across veterinary species.

### 4.5 Comparative analysis: context-dependent duality of miRNA functions across viral infections

A critical observation emerging from the literature is that several miRNAs—including miR-218, miR-122, miR-331-3p, miR-378, miR-24-3p, and miR-29a—exert opposing effects on viral replication depending on the infection context (Guo and Feng, 2025). This duality is not stochastic but arises from well-defined mechanistic determinants that can be categorized into four major axes (Fig. 2):

**Fig. 2:**
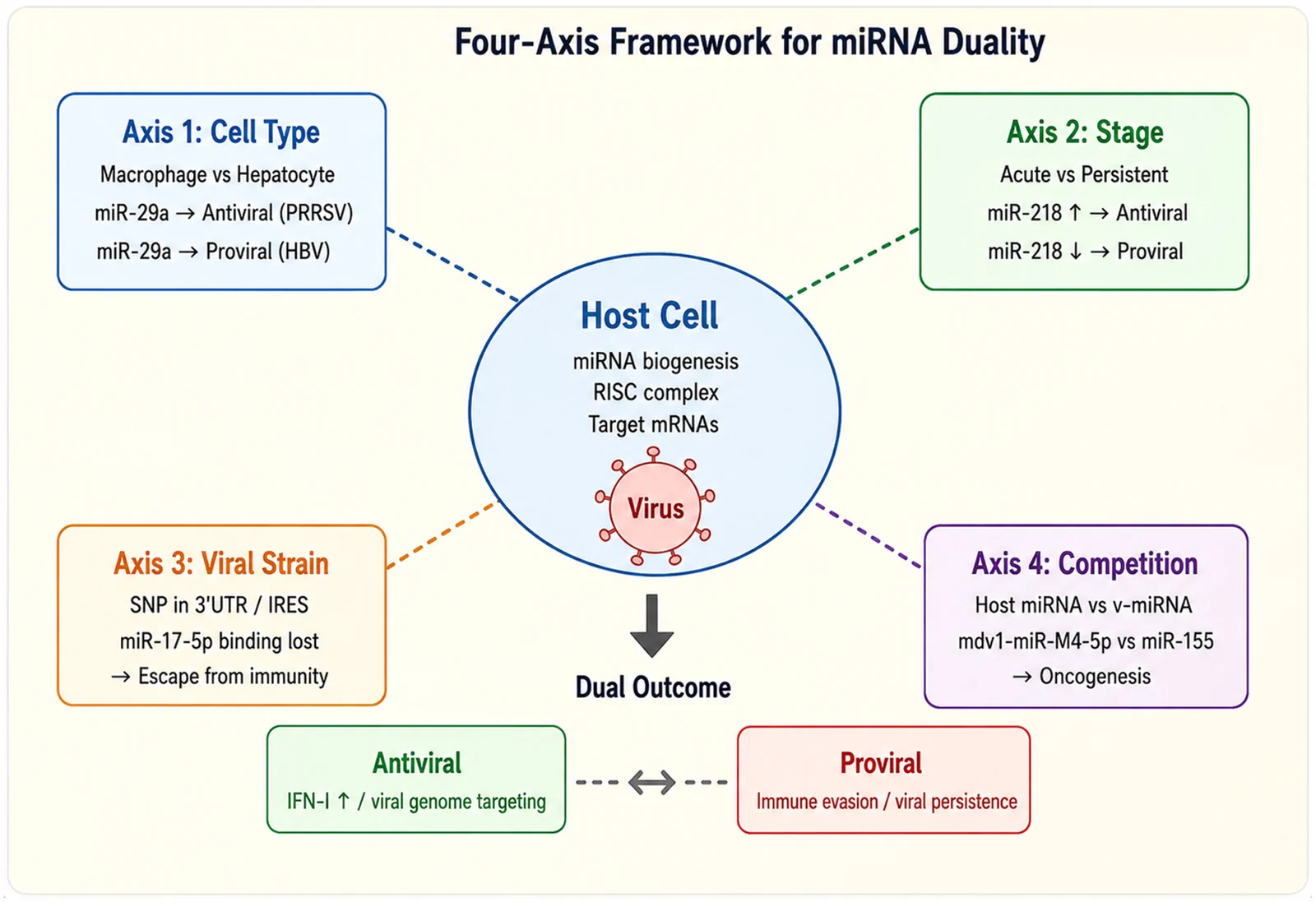
Four-Axis contextual framework explaining the antiviral versus proviral duality of MicroRNAs in Veterinary Viral Infections. This framework integrates four major determinants that govern the opposing functions of the same miRNA in different infection contexts. Axis 1 (Cell Type): cell-type-specific expression of co-factors (e.g., Ago proteins, RNA-binding proteins) determines miRNA loading and target accessibility; miR-29a suppresses PRRSV in macrophages but promotes HBV in hepatocytes. Axis 2 (Infection Stage): temporal dynamics during acute versus persistent infection alter miRNA expression and function; miR-218 is antiviral during acute PRRSV but downregulated during persistence. Axis 3 (Viral Strain): single nucleotide polymorphisms in viral 3′UTRs or IRES elements can abolish miRNA binding, enabling strain-specific escape (e.g., FMDV miR-17-5p resistance). Axis 4 (Host–Viral miRNA Competition): viral miRNAs (e.g., mdv1-miR-M4-5p) compete with host orthologs (gga-miR-155) for shared mRNA targets, shifting the balance toward viral persistence and oncogenesis. The net output—antiviral immunity versus proviral enhancement—emerges from the integration of these four axes, explaining why the same miRNA can exert opposite effects across different host–virus systems.

**Axis 1: Cell Type-Specific Expression of Co-Factors.** The availability of Argonaute proteins, RNA-binding proteins, and accessory factors varies considerably across cell types, directly influencing miRNA loading efficiency, strand selection, and RISC complex activity (Medley et al., 2021). For example, miR-29a suppresses PRRSV replication in porcine alveolar macrophages through targeting the viral Nsp2 gene, but promotes HBV replication in hepatocytes by downregulating the host restriction factor TRIM25 (Guo and Feng, 2025). This cell-type dependency highlights that the same miRNA can act as an antiviral or proviral factor purely based on the cellular context.

**Axis 2: Stage-Specific Dynamics During Infection.** The temporal phase of infection—acute, persistent, or latent—determines miRNA functionality. During acute PRRSV infection, miR-218 suppresses viral replication by enhancing type I interferon production. However, during persistent infection, the same miRNA becomes downregulated, allowing viral escape from immune surveillance (Zhang et al., 2026). This temporal switch suggests that miRNAs function as rheostats rather than simple on/off switches.

**Axis 3: Viral Strain-Specific Interactions.** Different strains of the same virus—particularly RNA viruses with high mutation rates—can evade or exploit miRNA targeting through single nucleotide polymorphisms in the 3′UTR of viral genomes. For instance, a single nucleotide variation in the FMDV IRES region abolishes miR-17-5p binding, converting a susceptible strain to a resistant one (Basagoudanavar et al., 2018). This explains why the same miRNA may appear antiviral in one outbreak but neutral or proviral in another.

**Axis 4: Competition Between Host and Viral miRNAs.** In herpesvirus infections such as MDV, viral miRNAs (e.g., mdv1-miR-M4-5p) compete with host miRNAs (gga-miR-155) for shared mRNA targets, effectively "hijacking" the regulatory network (Fotouh et al., 2026). This competition shifts the balance from host antiviral responses toward viral persistence and oncogenesis. The outcome depends on the relative abundance, binding affinity, and expression kinetics of host versus viral miRNAs.

**Synthesis and Implications:** The contextual duality of miRNA function is not a biological anomaly but a reflection of the sophisticated regulatory architecture of host-virus interactions. The four-axis framework proposed here (cell type, infection stage, viral strain, and host-viral miRNA competition) provides a novel, testable model for predicting miRNA behavior across different infection contexts—a contribution that distinguishes this review from prior descriptive summaries. Understanding the determinants outlined above is essential for: (a) predicting miRNA behavior in uncharacterized infection scenarios; (b) designing miRNA-based therapeutics with predictable outcomes; and (c) interpreting discrepant findings across studies. Future research should prioritize systematic mapping of these axes using standardized in vitro and in vivo models to establish predictive frameworks for miRNA function in specific infection contexts.

## 5 Circulating microRNAs as emerging biomarkers in veterinary medicine

### 5.1 Characteristics of Circulating miRNAs

Intracellular miRNAs are well recognized for their role in regulating a wide range of biological pathways. In addition to their intracellular functions, both immune and non-immune cells can release miRNAs into the extracellular environment through active secretion or passive release following membrane disruption (Miretti et al., 2020). These extracellular miRNAs are collectively referred to as circulating miRNAs (Miretti et al., 2020). The presence of circulating miRNAs in serum and plasma was first reported in 2008 (Mi et al., 2013), and subsequent studies identified them in various biological fluids, including whole blood, serum, plasma, tears, saliva, urine, cerebrospinal fluid, colostrum, and milk (Weber et al., 2010). Their active secretion into extracellular compartments supports the concept that circulating miRNAs may function as hormone-like mediators of intercellular communication (Correia et al., 2017).

One of the most notable characteristics of circulating miRNAs is their remarkable stability compared with other RNA species (Samir and Vaas, 2016). This property makes them highly suitable for use in archived biological samples, including specimens stored in biobanks (Samir and Vaas, 2016). Unlike messenger RNAs, miRNAs remain highly stable in formalin-fixed, paraffin-embedded tissue samples, allowing reliable assessment of their expression and localization even after long-term storage (Hall et al., 2012). Furthermore, circulating miRNAs exhibit exceptional resistance to harsh environmental conditions, including boiling, pH fluctuations, repeated freeze–thaw cycles, and prolonged storage (Brase et al., 2010; Glinge et al., 2017).

The stability of circulating miRNAs in body fluids is primarily attributed to two mechanisms: binding to protective proteins such as Argonaute 2 (Ago2), nucleophosmin 1 (NPM1), and high-density lipoproteins (HDL), forming stable miRNA–protein complexes; and encapsulation within extracellular vesicles, including exosomes, microvesicles, and apoptotic bodies (Iftikhar and Carney, 2016; Gallo et al., 2012).

### 5.2 miRNA biomarkers in veterinary viral diseases

In infectious diseases lacking efficient blood-based diagnostic approaches, miRNAs derived from alternative body fluids may provide valuable biomarker candidates (De Guire et al., 2013). The rapid alteration of certain circulating miRNAs in response to treatment makes them particularly suitable for therapeutic monitoring (De Guire et al., 2013). Their involvement in disease progression and treatment response further supports their potential as versatile biomarkers in veterinary medicine (De Guire et al., 2013).

**PRRSV Infection:** More than 30 miRNAs have been found to play a role in PRRSV infection (Zhang et al., 2026; Guo and Feng, 2025). The growing understanding of miRNA-mediated regulation has sparked interest in harnessing miRNAs as tools for antiviral therapy and prophylaxis, with applications extending to miRNA-guided diagnostic tools and miRNA-based live attenuated vaccines (Zhang et al., 2026; Guo and Feng, 2025).

**Foot-and-Mouth Disease:** Serum miRNA profiling has identified stage-specific markers—miR-1281 reduced during acute and persistent phases, bta-miR-17-5p elevated in acute phase, and bta-miR-31 elevated in persistent phase—offering potential for disease phase determination and subclinical carrier detection (Stenfeldt et al., 2017; Dong et al., 2017). Using miRNA PCR array plates, Stenfeldt and colleagues detected alterations in miRNA levels in bovine serum samples collected from three different phases of FMDV infection, identifying a total of 169 abundant miRNAs and 3 differentially expressed miRNAs (Stenfeldt et al., 2017).

**Ebola Virus Disease:** The identification of an Ebola virus-derived miRNA-like fragment (miR-vp-3p) detectable before viral genomic RNA appears in serum highlights the potential of viral miRNA-like fragments as early biomarkers (Chen et al., 2016). Chen and colleagues identified this fragment in the serum of Ebola patients, demonstrating its potential utility for early diagnosis of Ebola virus disease (Chen et al., 2016).

**Bovine Viral Diarrhea:** Taxis and colleagues investigated circulating miRNA profiles in BVDV-infected calves and identified differential expression patterns for bta-miR-151-3p and bta-miR-423-5p, although neither was considered a reliable diagnostic biomarker due to insufficient consistency and specificity (Taxis et al., 2017).

**Hendra Virus:** Circulating miR-16 and miR-32 were validated as promising biomarker candidates for HeV infection in horses, with miRNA expression ratios demonstrating improved discrimination between infected and healthy horses (Cowled et al., 2017).

### 5.3 Technical considerations and pre-analytical factors

Despite their promise, several pre-analytical factors can substantially affect circulating miRNA quality and quantification (McDonald et al., 2011; Blondal et al., 2013). Sample collection, processing, and storage conditions are essential for ensuring reliable results (McDonald et al., 2011; Blondal et al., 2013). Critical considerations include:

• **Anticoagulant selection:** Heparin inhibits PCR amplification and should be avoided; blood samples should be collected in EDTA-coated tubes (Kim et al., 2012; Li et al., 2016)
• **Hemolysis:** Must be strictly minimized, as intracellular miRNAs released from erythrocytes and platelets can significantly alter circulating miRNA profiles (Pritchard et al., 2012). The expression ratio of miR-451 (erythrocyte-specific) to miR-23a has been proposed as a practical indicator for detecting hemolysis (Blondal et al., 2013)
• **Extraction methods:** Different commercial kits can yield varying results. A study by Guo and colleagues compared five kits for isolating circulating extracellular sRNAs, finding that Ambion TRIzol LS produced the most reads for miRNAs, while the QiaSymphony RNA extraction kit showed the highest repeatability (Guo et al., 2017)
• **Detection platforms:** Current technologies include stem-loop reverse transcription followed by TaqMan PCR analysis, poly(A)-tailed RT-qPCR, miRNA microarrays, and next-generation sequencing (miRNA-seq) (Ho et al., 2022)
• **Normalization methods:** The choice of internal controls, spike-ins, and normalization methods can significantly influence miRNA expression patterns (Blondal et al., 2013)

### 5.4 Biomarker development pipeline

Biomarker development generally involves three major stages: discovery, validation, and translation (Do et al., 2021). The discovery phase focuses on identifying miRNAs with potential biomarker value. The validation phase involves confirming biomarker reliability across diverse populations and sample sets. Finally, the translation phase aims to integrate validated biomarkers into practical clinical applications. This pipeline is described here because it provides the conceptual framework against which the current progress of veterinary miRNA biomarkers can be evaluated (see Sections 6.5 and 9.4). To date, most miRNA studies in veterinary medicine have primarily focused on identifying differential miRNA expression, corresponding mainly to the discovery stage (Do et al., 2021). Progressing candidate biomarkers through subsequent stages of development is essential for routine clinical application.

**Key Challenges in Clinical Translation:**

• Biological variation: miRNA expression profiles can be influenced by age, sex, breed, physiological status, and environmental conditions (Do et al., 2021)
• Standardization: Variability in sample collection, RNA extraction methods, detection platforms, and normalization strategies leads to inconsistencies (Do et al., 2021)
• Species-specific databases: Many domestic animal species lack well-annotated reference genomes and comprehensive miRNA databases. While 962 miRNAs have been annotated for dogs in miRBase, no miRNAs from cats have been annotated, highlighting a significant gap in companion animal miRNA research (Ojo and Kreuzer-Redmer, 2023)

## 6 MicroRNA-based therapeutics and vaccines

This section addresses research question (4) of the conceptual framework: the translational barriers to clinical implementation and how they can be overcome (Dimension c: translational applications). Fig. 3 (presented before Section 10) provides a decision framework for selecting and prioritising miRNA-based strategies by Technology Readiness Level (TRL), viral genome type, and intended application; each strategy discussed below is cross-referenced to its current TRL estimate.

**Fig. 3:**
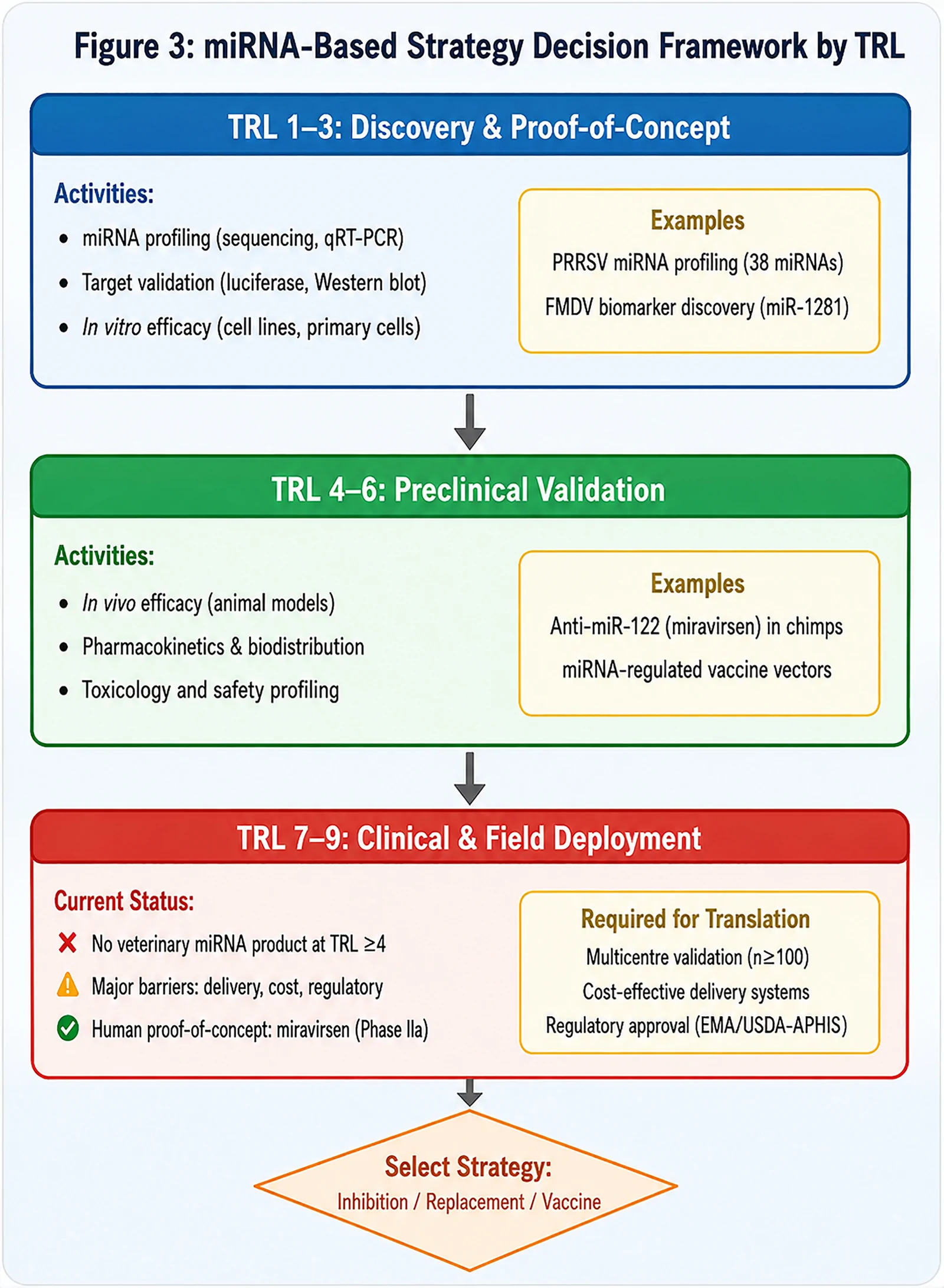
Technology readiness level (TRL) decision framework for selecting miRNA-based strategies in veterinary viral diseases. This decision framework guides the selection of miRNA-based strategies according to their Technology Readiness Level (TRL). At TRL 1–3 (Discovery Phase), the primary activities are miRNA profiling, target identification, and in vitro validation; examples include PRRSV miRNA screening and FMDV biomarker discovery. At TRL 4–6 (Preclinical Validation Phase), in vivo efficacy, pharmacokinetics, and toxicology are evaluated; notable examples include anti-miR-122 (miravirsen) in chimpanzees and miRNA-regulated vaccine vectors. At TRL 7–9 (Clinical Translation Phase), the framework highlights that currently no veterinary miRNA product has reached TRL ≥4, with major barriers including delivery systems, cost-effectiveness, and regulatory pathways. The decision diamond at the base prompts selection among inhibition (antagomirs/LNA-ASOs), replacement (miRNA mimics), or vaccine adjuvant strategies, based on the specific viral pathogen, host species, and intended application. This framework emphasizes that most veterinary miRNA research remains in early stages, and that multicentre validation (n≥100 per group) and standardized protocols are critical for progression to clinical deployment.

### 6.1 Therapeutic strategies: inhibition and replacement

Synthetic miRNA-based therapeutics are generally developed using two principal approaches: miRNA inhibition and miRNA replacement (Shah and Calin, 2014; Bernardo et al., 2015).

**miRNA Inhibition Strategy:** Aims to suppress disease-associated miRNAs whose overexpression contributes to pathological conditions (Bernardo et al., 2015). Key tools include:

• **Antagomirs** and **LNA-modified antisense oligonucleotides**: Function as antisense oligonucleotides that bind complementarily to target miRNAs, preventing their interaction with target mRNAs (Lima et al., 2018; Stenvang and Kauppinen, 2008)
• **miRNA sponges**: Plasmid-based constructs containing multiple binding sites for one or more miRNAs, capable of simultaneously sequestering multiple miRNAs (Ebert and Sharp, 2010)
• **miRNA Replacement Strategy:** Focuses on restoring beneficial miRNAs that are downregulated or functionally impaired (Bernardo et al., 2015). Principal tools include:
• **miRNA mimics**: Synthetic double-stranded RNA molecules designed to replicate endogenous mature miRNAs (Bader et al., 2010)
• **DNA plasmids** encoding miRNA sequences (Do et al., 2021)

### 6.2 Antiviral applications

Beyond their established therapeutic applications in cancer and genetic disorders, miRNAs are now recognized as key molecular orchestrators of host–virus interaction (Bernier and Sagan, 2018). Differentially expressed host miRNAs and virus-encoded miRNAs can be selectively targeted using synthetic antiviral molecules, offering novel opportunities for precision antiviral therapies (Samir and Pessler, 2016; Arbuthnot, 2011).

**PRRSV:** The growing understanding of miRNA-mediated regulation has sparked interest in harnessing miRNAs as tools for antiviral therapy and prophylaxis, with advances in synthetic miRNA-based antiviral therapeutics, miRNA-guided diagnostic biomarkers, and miRNA-based live attenuated vaccines (Zhang et al., 2026; Guo and Feng, 2025).

**Foot-and-Mouth Disease:** A dual-miRNA construct containing two hairpin structures targeting the viral IRES demonstrated significantly greater inhibition of viral replication in vitro compared with conventional miRNA sequences (Chang et al., 2014), highlighting the potential of combinatorial miRNA-based antiviral approaches.

**Infectious Bursal Disease:** Targeting viral VP1 and VP2 genes using miRNAs delivered through a recombinant avian adeno-associated virus vector effectively suppressed IBDV replication in 8-day-old SPF chickens (Shen et al., 2011).

**Avian Leukosis Virus:** miRNA-based therapeutics, including mimics and inhibitors targeting regulatory miRNAs, represent a promising platform for the development of targeted antiviral interventions (Fotouh et al., 2026).

### 6.3 Vaccine development and adjuvant applications

miRNAs regulate multiple components of both innate and adaptive immune responses, including antibody production, cytokine secretion, inflammation, and immune cell differentiation (Pedersen and David, 2008). Experimental studies have demonstrated that vaccination can alter miRNA expression profiles in immune-related tissues. Given their broad regulatory effects on immune signaling pathways, miRNAs have emerged as promising candidates for vaccine adjuvant development (Mochida and Uchida, 2024). These molecules may be administered alone or alongside antigens, commonly through nanoparticle-based delivery systems (Mochida and Uchida, 2024). miRNA-regulated gene delivery systems incorporating miRNA target sites can restrict transgene expression in off-target tissues, improving vector safety (Dhungel et al., 2018). This strategy has demonstrated effectiveness in viral systems including poliovirus, influenza A virus, flaviviruses, and dengue virus (Dhungel et al., 2018).

A well-characterised example from human vaccinology illustrates the mechanistic principle: insertion of multiple copies of a miR-142-3p target sequence into the 3′ UTR of a yellow fever virus-based flavivirus vector prevented haematopoietic-cell tropism, attenuating systemic spread while preserving immunogenicity at the site of inoculation (Proutski et al., 1997). Translating this strategy to veterinary pathogens such as PRRSV would require: (1) identification of a tissue-restricted host miRNA with stable, high expression specifically in the cell types whose infection drives pathogenesis (e.g., alveolar macrophages); (2) insertion of the corresponding miRNA target sequence into the viral genome without disrupting replication fitness; and (3) verification that mutational escape does not occur under field selective pressure, given PRRSV’s high mutation rate (Zhang et al., 2026).

#### 6.3.1 Immunological safety considerations for miRNA-based vaccine components

Although miRNAs do not encode proteins and are therefore non-immunogenic in the classical sense, the single-stranded and double-stranded RNA intermediates generated during their delivery or processing can activate endosomal Toll-like receptors—particularly TLR3, TLR7, and TLR8—leading to innate immune activation and, in some contexts, pro-inflammatory cytokine release (Hornung et al., 2005). Because miRNAs also regulate self-tolerance networks (e.g., miR-146a in FOXP3+ regulatory T-cell differentiation), perturbation of these pathways carries a theoretical risk of breaking self-tolerance and promoting autoimmune pathology, a concern that warrants systematic assessment in the relevant veterinary target species (Eberle et al., 2008). Chemical modifications substantially mitigate immunostimulatory risk: 2ʹ-O-methyl modification of the RNA backbone reduces TLR7/8 recognition; phosphorothioate linkages confer nuclease resistance without substantially increasing immunogenicity; and locked nucleic acid (LNA) chemistry improves binding affinity, potentially permitting lower doses that further limit non-specific immune activation (Eberle et al., 2008; Hornung et al., 2005). Species-specific TLR expression profiles and target-tissue immunology must be characterised before any miRNA-adjuvant formulation is advanced to in vivo studies in the relevant veterinary species.

### 6.4 Delivery systems and challenges

The clinical success of miRNA-based therapeutics depends heavily on safe, efficient, and targeted delivery systems capable of preventing degradation, minimizing rapid clearance from circulation, and enhancing cellular uptake (Holjencin and Jakymiw, 2022). Various delivery platforms have been investigated, including nanoparticle-based systems, viral vectors, exosomes, and electroporation (Taghdiri and Mussolino, 2024).

**Key Translational Challenges:**

• Nuclease-mediated degradation and rapid systemic clearance (Eberle et al., 2008; Hornung et al., 2005)
• Off-target effects and passenger-strand loading leading to unintended interactions (Søkilde et al., 2015)
• Inefficient tissue-specific delivery
• Saturation of endogenous miRNA processing machinery, leading to toxicity and pathology (Kappari et al., 2024)
• Practical considerations for delivery to large groups of animals: individual administration may be feasible for high-value animals (e.g., racehorses, SPF parent flocks) but impractical for mass administration in production settings (Kappari et al., 2024)

Practical considerations for delivering to large groups of animals: individual administration may be feasible for high-value animals (e.g., racehorses, breeding bulls, SPF parent flocks) but is economically and logistically impractical for mass-reared production animals. Oral delivery is particularly challenging for ruminants because the highly acidic rumen environment and the multi-compartment digestive system rapidly degrade unprotected RNA. Polymer- or lipid-based microparticle encapsulation and pH-stable lipid nanoparticles are active areas of development for ruminant oral delivery of nucleic acid therapeutics, although none has yet reached veterinary regulatory approval. For poultry, in-ovo injection during embryonation and spray-based intranasal delivery have been explored as scalable routes, but miRNA stability under farm environmental conditions (temperature fluctuations, UV exposure) and batch-to-batch consistency remain unresolved challenges. For aquaculture species, bath immersion with nanoparticle-encapsulated miRNA constructs has been trialed for short-duration challenges in fish, but dilution effects in open-water systems make therapeutic dosing nearly infeasible under production conditions; bath delivery is more plausible for contained recirculating aquaculture systems (RAS).

### 6.5 Current translational status and lessons from early validation efforts

Despite substantial preclinical interest, most miRNA-based candidates for veterinary viral diseases remain at the discovery or early validation stage; to date, no miRNA-based diagnostic or therapeutic product for a veterinary viral disease has reached routine clinical or field deployment (Do et al., 2021). Reviewing the completed validation studies—both encouraging and inconclusive—helps clarify the specific barriers that must be overcome before translation is realistic.

#### 6.5.1 Encouraging proof-of-concept findings

Stenfeldt and colleagues demonstrated, in a controlled challenge study, that serum bta-miR-17-5p and bta-miR-31 tracked distinct phases of FMDV infection in cattle, providing proof-of-concept evidence for stage-specific biomarkers capable of distinguishing acute infection from the persistent carrier state (Stenfeldt et al., 2017); whether this signature performs with adequate sensitivity and specificity in naturally infected, genetically diverse field populations has not yet been reported. Similarly, Cowled and colleagues validated circulating miR-16 and miR-32 as candidate biomarkers that discriminate HeV-infected from healthy horses in an experimental infection model, with expression ratios improving diagnostic discrimination (Cowled et al., 2017); prospective validation in naturally exposed horses remains an important next step. More broadly, the principle that a host miRNA essential to a virus’s life cycle can be pharmacologically silenced to achieve durable antiviral suppression was first established outside veterinary medicine, with the anti-miR-122 compound miravirsen: sustained silencing of miR-122 produced a prolonged, resistance-free reduction in hepatitis C viral load in chronically infected chimpanzees (Lanford et al., 2010), a finding that was subsequently translated into human Phase I/IIa trials. Because miR-122 also has a central, conserved role in host lipid metabolism, any future attempt to apply an analogous host-miRNA-silencing strategy to a veterinary pathogen would require dedicated, species-specific toxicology studies rather than direct extrapolation from the human experience.

#### 6.5.2 Inconclusive validation findings

Not all candidate biomarkers have withstood validation. Taxis and colleagues identified differential expression of bta-miR-151-3p and bta-miR-423-5p in the serum of BVDV-challenged calves, but concluded that neither miRNA met the consistency and specificity required to serve as a reliable diagnostic biomarker (Taxis et al., 2017). This outcome illustrates a broader pattern in the field: candidate miRNAs identified in single-cohort discovery studies frequently do not retain diagnostic performance when re-examined, underscoring the need for independent, multi-cohort validation before any biomarker claim is accepted.

#### 6.5.3 Lessons for clinical translation

Taken together, these completed studies point to several critical success factors for future translational work:

**Multi-center validation:** independent replication using standardized sample-collection and analytical protocols to reduce inter-laboratory variability (Do et al., 2021; Blondal et al., 2013).

**Cost-effectiveness:** candidate biomarker panels and therapeutics must be economically competitive with existing diagnostic or therapeutic standards, since veterinary budgets—particularly in production-animal settings—are far more constrained than in human medicine.

**Species-specific safety and efficacy profiling:** a conserved miRNA’s physiological role in the target species cannot be assumed from data obtained in humans or laboratory animals and must be independently established.

**Stakeholder engagement:** early involvement of veterinary practitioners, diagnostic laboratories, and regulatory agencies is essential for defining realistic performance requirements and regulatory pathways.

None of the candidates reviewed above has yet progressed through all of these stages, consistent with the discovery-phase status of the field described in Section 5.4 (Do et al., 2021).

## 7 MicroRNA in specific veterinary viral diseases

### 7.1 Porcine reproductive and respiratory syndrome (PRRS)

PRRSV continues to pose a major and persistent threat to the global swine industry, largely owing to its high mutation rate and ability to evade host immune responses (Zhang et al., 2026). A wealth of studies has explored the RNA-level interplay between the host and PRRSV, identifying 38 miRNAs involved in infection to date (Zhang et al., 2026; Guo and Feng, 2025). Among these, 27 exhibit antiviral properties, while 11 promote viral replication (Zhang et al., 2026; Guo and Feng, 2025).

**Antiviral miRNAs** suppress PRRSV replication through diverse mechanisms:

• Targeting viral genomes directly (Zhang et al., 2026)
• Modulating entry receptor expression (Zhang et al., 2026)
• Regulating type I interferon (IFN-I) and NF-κB signaling pathways (Guo and Feng, 2025)
• Enhancing innate immune responses (Guo and Feng, 2025)

**Proviral miRNAs** facilitate viral replication by:

• Suppressing antiviral immune responses (Guo and Feng, 2025)
• Modulating host factors essential for viral replication (Guo and Feng, 2025)
• Creating cellular environments permissive for viral propagation (Guo and Feng, 2025)

The context-dependent duality of miRNA regulation—where the same miRNA can exert opposite effects in different viral infection contexts—underscores the complexity of miRNA-mediated regulation and highlights the importance of understanding cell type-specific and stage-specific effects (Guo and Feng, 2025; Table 1). This growing understanding has sparked interest in miRNA-based antiviral therapeutics, diagnostic biomarkers, and live attenuated vaccines for PRRSV control (Zhang et al., 2026; Guo and Feng, 2025).

**Table 1:** Candidate miRNA biomarkers and functional miRNAs in PRRS and Marek's disease: expression direction, validated targets, and validation status. Direction refers to infected vs. uninfected animals in the principal cited study. Validation status: qRT-PCR = expression confirmed; in vitro = target validated in cell culture; luciferase = 3′UTR luciferase reporter assay; KO virus = confirmed by viral knockout or mutagenesis.

**miRNA**	**Disease**	**Direction in infected animals**	**Target / Pathway**	**Validation status**
ssc-miR-146a-3p	PRRSV	Up in serum	NF-κB signalling; TRAF6	qRT-PCR, in vitro
ssc-miR-21-3p	PRRSV	Up in serum/PBMC	PDCD4; anti-apoptotic	qRT-PCR, in vitro
ssc-miR-23a	PRRSV	Down in lung	IFN-β signalling	qRT-PCR
ssc-miR-30c	PRRSV	Down in serum	Viral entry (SMPD3)	qRT-PCR, luciferase
ssc-miR-125a	PRRSV	Up in serum	IRF4; innate immune modulation	qRT-PCR
gga-miR-21	MDV	Up in tumor tissue	PDCD4; anti-apoptotic	qRT-PCR, in vitro (Stik et al., 2013)
mdv1-miR-M4-5p (v-miRNA)	MDV	Virus-encoded; expressed in tumours	Functional ortholog of gga-miR-155; indispensable for lymphomagenesis	KO virus, in vivo (Bondada et al., 2019)
gga-miR-155	MDV	Up in tumor/PBMC	CD3ζ; TCR signalling; T-cell expansion	qRT-PCR
gga-miR-26a	MDV	Down in spleen during tumorigenesis	Cyclin D2	qRT-PCR (Li et al., 2014a)
gga-miR-103-3p	MDV	Down in splenic/hepatic tumors	Cyclin E1; DP-2	qRT-PCR (Han et al., 2016)

Despite the growing number of PRRSV miRNA studies, direct comparisons between them are constrained by substantial inter-study heterogeneity. Key sources of variation include differences in viral strain (Type I/European vs. Type II/North American, and their numerous circulating sub-lineages), host genetics (different commercial pig lines vary in innate immune receptor expression), animal age and immune status at the time of infection, and sample type (bronchoalveolar lavage fluid vs. serum vs. PBMC). These factors routinely produce divergent or even contradictory miRNA expression results across studies, and future work should explicitly report these variables to enable meaningful meta-analyses (Do et al., 2021; Guo and Feng, 2025).

### 7.2 Marek's disease (MD)

Marek's disease, caused by Gallid herpesvirus 2 (MDV), is a major viral neoplastic disease in poultry associated with annual economic losses exceeding USD 1–2 billion globally (Fotouh et al., 2026). MDV represents a unique case in which a viral microRNA ortholog (mdv1-miR-M4-5p) is indispensable for tumor induction in vivo—the first direct demonstration of a virus-encoded miRNA acting as a primary oncogenic factor in a natural host (Fotouh et al., 2026; Kappari et al., 2024; Bondada et al., 2019). This viral miRNA is a functional ortholog of gga-miR-155, and the miR-155 pathway has been shown to be critical for the induction of MDV-induced lymphomas (Bondada et al., 2019).

**Host miRNA Involvement in MDV Pathogenesis:**

• **gga-miR-21**: Significantly elevated in virulent strain infection, promotes tumor formation through repression of programmed cell death 4 (PDCD4) (Stik et al., 2013)
• **miR-26a**: Significantly reduced in the spleen during tumorigenesis (Li et al., 2014a)
• **miR-103-3p**: Decreased expression in splenic and hepatic tumors; under normal conditions suppresses cell migration and tumorigenesis by targeting Cyclin E1 and transcription factor DP-2 (Han et al., 2016)
• **miR-219b**: Promotes programmed cell death through regulation of apoptotic pathways (Zhao et al., 2017)

The integration of host miRNA expression profiles into molecular surveillance programs may improve early identification of emerging pathogenic MDV strains while supporting rational design and evaluation of next-generation vaccines (Fotouh et al., 2026).

Comparability of MDV miRNA studies is also complicated by inter-study heterogeneity, including differences in MDV pathotype (very virulent plus vs. classical virulent), host breed (Leghorn vs. broiler vs. layer), vaccination status, and tissue sampled (T-cell tumour vs. peripheral blood vs. feather follicle). As for PRRSV, systematic reporting of these variables is a prerequisite for any future meta-analytic synthesis (Fotouh et al., 2026; Bondada et al., 2019).

### 7.3 Avian leukosis virus (ALV)

ALV, a member of the genus *Alpharetrovirus*, causes a variety of neoplastic diseases in birds, including B-cell lymphoma, hemangioma, and myelocytoma (Payne and Nair, 2012). Unlike MDV, which encodes its own oncogenic miRNA, ALV exploits host miRNA pathways through insertional mutagenesis and NF-κB-mediated transcriptional activation (Fotouh et al., 2026).

**Key Findings:**

• **gga-miR-221 and gga-miR-222**: Significantly upregulated in infected livers, contribute to tumorigenesis by targeting BCL-2-related regulatory pathways (Dai et al., 2015)
• **gga-miR-375**: Downregulated in ALV-J infection; overexpression inhibits YAP1, Cyclin E (CCNE1), and DIAP1, suppressing proliferation of DF-1 chicken fibroblasts (Li et al., 2014b)
• **miR-23**: Promotes viral replication in the spleen by downregulating interferon regulatory factor 1 (IRF1)
• **miR-34b-5p**: Enhances ALV-J replication through inhibition of melanoma differentiation-associated gene 5 (MDA5) signaling (Li et al., 2017)

The ALV-induced activation of the miR-155 pathway has been well documented, with ALV activating miR-155 by insertional activation (Bondada et al., 2019). These observations collectively indicate that miRNAs function as central regulators of host–virus interactions by coordinating viral replication and oncogenic signaling pathways, highlighting host miRNA networks as promising therapeutic targets (Fotouh et al., 2026).

### 7.4 Infectious bursal disease (IBD; gumboro disease)

IBD, caused by infectious bursal disease virus (IBDV), is a highly contagious immunosuppressive disease affecting young chickens (Mahgoub, 2012). Interactions between miRNAs and IBDV reveal complex regulatory mechanisms involving both viral replication and host immune responses.

**Key miRNA–IBDV Interactions:**

• gga-miR-9*: Induced by IBDV infection, promotes viral replication by suppressing type I interferon production through targeting of interferon regulatory factor 2 (IRF2) (Ouyang et al., 2015)
• **gga-miR-2127-3p**: Weakens host antiviral responses by downregulating translation of chicken p53 (Ouyang et al., 2017)
• **gga-miR-130b-3p**: Inhibits viral replication by directly targeting both the viral genome and the suppressor of cytokine signaling 5 (SOCS5) gene (Fu et al., 2017)

The ability of miRNAs to selectively modulate both viral and host targets provides a promising foundation for the development of precision antiviral strategies, though improvements in target specificity, delivery technologies, and safety profiles are still required.

### 7.5 Foot-and-mouth disease (FMD)

FMD is a highly contagious viral disease affecting cloven-hoofed animals, causing substantial economic burdens (Alexandersen and Mowat, 2005). miRNA investigations have identified 39 significantly upregulated miRNAs in the serum of cattle infected with FMDV serotype O, nine of which exhibited sequence similarity to the viral genome (Basagoudanavar et al., 2018).

**Stage-Specific Biomarkers:**

• **miR-1281**: Significantly reduced during both acute and persistent phases (Stenfeldt et al., 2017)
• **bta-miR-17-5p**: Highest expression during acute phase (Stenfeldt et al., 2017)
• **bta-miR-31**: Highest expression during persistent phase (Stenfeldt et al., 2017)

These dynamic changes indicate that serum miRNA profiling may offer a promising approach for disease phase determination and identifying subclinically infected FMDV carriers (Stenfeldt et al., 2017; Dong et al., 2017). Stenfeldt and colleagues detected a total of 169 abundant miRNAs in serum samples and identified 3 differentially expressed miRNAs, noting that cattle that cleared infection resembled the baseline profile (Stenfeldt et al., 2017). However, the high genetic variability of FMDV and existence of multiple serotypes remain major challenges for developing universally effective antiviral therapies (Kappari et al., 2024).

### 7.6 Ebola virus disease (EVD)

EVD is a severe zoonotic hemorrhagic fever associated with high mortality rates. Early detection is essential for improving treatment outcomes and preventing transmission. Chen and colleagues identified an Ebola virus-derived miRNA-like fragment, miR-vp-3p, detectable before viral genomic RNA appeared in serum samples, highlighting its potential utility as an early biomarker (Chen et al., 2016).

A separate study by Duy and colleagues identified eight host miRNAs (including hsa-miR-146a-5p, hsa-miR-21-3p, hsa-miR-22-3p, and hsa-miR-29a-3p) as potential classifiers of Ebola virus infection, correctly distinguishing infection status in approximately 86% of human cases and detecting infection-related changes before clinical symptom onset in nearly half of macaques (Duy et al., 2016).

The clinical utility of miRNA biomarkers in EVD extends beyond early detection. A longitudinal study by Duy and colleagues demonstrated that hsa-miR-146a-5p and hsa-miR-21-3p could distinguish fatal from non-fatal outcomes with 78% accuracy when measured within 48 hours of symptom onset (Duy et al., 2016). Mechanistically, miR-146a-5p targets TRAF6 and IRAK1, dampening NF-κB-mediated pro-inflammatory responses—a phenomenon that may explain the paradoxical immune suppression observed in severe Ebola cases. Furthermore, the Ebola virus-encoded miR-vp-3p has been shown to downregulate host SOCS1, enhancing viral replication while simultaneously reducing the sensitivity of diagnostic RT-PCR assays (Chen et al., 2016). This dual role highlights the need to consider both host and viral miRNAs when developing diagnostic algorithms.

### 7.7 Hendra virus infection (HeV)

HeV is an emerging zoonotic pathogen causing severe disease in horses and humans. Cowled and colleagues identified 37 significantly differentially expressed miRNAs in infected horses, with miR-16 and miR-32 validated by qRT-PCR as promising biomarker candidates (Cowled et al., 2017). Analysis of miRNA expression ratios demonstrated improved discrimination between infected and healthy horses (Cowled et al., 2017). The development of diagnostic tools capable of identifying infected horses during the preclinical stage could substantially reduce the risk of zoonotic transmission during outbreak management (Cowled et al., 2017).

### 7.8 Rabies

Rabies is a highly lethal neurotropic viral disease affecting the central nervous system (Knobel et al., 2005). Recent studies have shown that brain miRNA expression profiles in RV-infected mice differ significantly from those of uninfected controls. Zhao and colleagues identified ten upregulated miRNAs and six downregulated miRNAs in the brains of infected mice (Zhao et al., 2012). Altered expression of these miRNAs appears to influence immune-related signaling pathways and may contribute to the pathological manifestations of infection (Zhao et al., 2012).

In addition, miR-133, which is specifically expressed in skeletal muscle, was predicted to bind to the N (nucleocapsid) and G (glycoprotein) transcripts of rabies virus (Samir and Vaas, 2016). This interaction may contribute to the prolonged dormant phase of the virus in skeletal muscle during the early stage of infection, prior to its progression into the nervous system (Samir and Vaas, 2016).

The functional consequences of these miRNA changes for blood–brain barrier integrity and for post-vaccination immune monitoring remain to be established and represent an underexplored area for future veterinary miRNA research.

### 7.9 Viral diseases in aquatic species

While most veterinary miRNA research has focused on terrestrial livestock and poultry, emerging evidence indicates that miRNAs also participate in viral diseases affecting aquaculture species. Viral hemorrhagic septicemia virus (VHSV) infection strongly upregulates the teleost-specific miR-462/miR-731 cluster in the liver of rainbow trout; this cluster carries interferon-inducible promoter elements and is similarly induced by the viral RNA mimic poly I:C, pointing to a role in the antiviral interferon response rather than a housekeeping function (Schyth et al., 2015). Similarly, infectious hematopoietic necrosis virus (IHNV) infection of rainbow trout alters expression of miR-146a-3p, miR-155-5p, miR-216a-5p, and miR-499b-5p, with miR-146a-3p and miR-216a-5p shown to dampen the type I interferon and Mx1 antiviral response, consistent with a proviral, immune-suppressive role for these two miRNAs during IHNV infection (Cao et al., 2018).

The application of miRNA-based diagnostics in aquatic settings faces unique challenges, including temperature-dependent miRNA stability, dilution effects in environmental samples, and limited species-specific reference databases. Given the substantial economic importance of global aquaculture and the recurring threat of viral outbreaks in farmed fish and shellfish, further investment in miRNA research for aquatic animal health is warranted.

Beyond salmonid rhabdoviruses, miRNA responses have also been documented in two economically important diseases of cultivated fish and shellfish. In common carp (Cyprinus carpio) infected with Cyprinid herpesvirus 3 (CyHV-3; Koi herpesvirus), RNA-seq analysis identified 29 differentially expressed host miRNAs, with several predicted to target immune-related mRNAs including interferon regulatory factors; notably, a distinct cohort of miRNAs was upregulated in the gills—the primary site of CyHV-3 replication—compared with spleen, suggesting tissue-specific regulation of the antiviral response (Donohoe et al., 2015). In Pacific white shrimp (Penaeus vannamei) challenged with White Spot Syndrome Virus (WSSV), the haemolymph miRNA profile shifts markedly, with miR-100 targeting the WSSV-encoded IE1 gene to suppress viral gene expression, and with miR-7 downregulation releasing its normal suppression of the STAT signalling pathway, thereby contributing to immune activation (Wang and Zhu, 2017). These findings establish that miRNA-mediated host–virus interactions extend across the major aquaculture taxa (teleost fish, cyprinid herpesvirus hosts, and crustaceans), and underscore the potential value of miRNA-based surveillance in aquatic animal health, where temperature-dependent miRNA stability, dilution in environmental samples, and the limited annotation of non-salmonid small-RNA databases present additional challenges compared with terrestrial livestock.

### 7.10 Cross-disease comparative analysis: convergent miRNA signatures

Across the disease-specific sections above, two miRNAs emerge as particularly recurrent: miR-155 and miR-146a. miR-155 (and its avian ortholog gga-miR-155) is upregulated in MDV-induced lymphoma, ALV-associated transformation, and IBDV-infected bursal tissue, consistently targeting PDCD4 and CD3ζ to promote cell survival and dampen TCR signalling (Fotouh et al., 2026; Bondada et al., 2019; Stik et al., 2013). In PRRSV infection, miR-155 upregulation in macrophages suppresses SHIP-1 and SOCS1, potentiating pro-inflammatory cytokine production (Guo and Feng, 2025)—a proviral outcome via immunopathology rather than direct replication support. This disease-specific divergence despite conserved miRNA upregulation illustrates why the four-axis framework (Section 4.5 / Fig. 2) is needed: the same miRNA, in different cell types and viral contexts, produces opposite net outcomes. miR-146a shows a complementary pattern: upregulated as a negative-feedback regulator of NF-κB signalling in PRRSV, IBDV, and Ebola infection, it acts primarily as an antiviral brake on excessive inflammation; however, MDV-infected T-cells appear to exploit its TRAF6-suppressing activity to evade cytotoxic immune clearance (Duy et al., 2016; Guo and Feng, 2025). These convergent yet context-dependent roles identify miR-155 and miR-146a as high-priority candidates for multi-disease biomarker panel development and cross-species One Health translation (Table 2). The hypothesis that this convergence reflects shared host signaling pathway targeting is discussed in Section 8.3.

**Table 2:** Selected miRNAs with conserved host-pathogen interaction roles across human and veterinary viral diseases, supporting One Health biomarker and therapeutic development. Seed-region conservation noted where sequence identity is documented.

**miRNA**	**Human viral disease context**	**Veterinary viral disease context**	**Conservation note**
miR-146a	HIV, HCV, SARS-CoV-2 (NF-κB suppression)	PRRSV, IBDV (innate immune modulation)	Highly conserved; human sequence identical to ssc/gga homologue at seed region
miR-155	EBV-induced lymphomagenesis	MDV lymphomagenesis (gga-miR-155; mdv1-miR-M4-5p ortholog)	Oncomiric role conserved across mammalian and avian herpesviruses
miR-21	HCV, HIV (anti-apoptotic)	PRRSV, MDV (PDCD4 suppression, pro-survival)	Seed-region sequence identical across vertebrates
miR-122	HCV (essential host factor for replication)	Conserved in livestock; BVDV interaction under investigation	Target of miravirsen (clinical anti-HCV agent)
miR-223	Influenza A (neutrophil regulation)	Influenza A in swine/avian hosts	Sequence conserved; cross-species biomarker potential
let-7 family	Herpes simplex, influenza (antiviral via HMGA2)	MDV, PRRSV (multiple targets)	Pan-vertebrate conservation; multiple family members

## 8 Emerging frontiers and novel perspectives

### 8.1 Viral seed mimics and sponges

Recent computational analyses have revealed that viruses can harbor sequence motifs that either mimic host miRNAs (viral seed mimics, vSMs) or inhibit their function (viral seed sponges, vSSs) (Mears and Bakre, 2024). Studies on Newcastle disease virus demonstrated that virulent strains encode a statistically significantly higher number of vSMs and a lower number of vSSs relative to low-virulence strains, suggesting that the capacity to interact with host miRNAs is a determinant of viral pathogenicity (Mears and Bakre, 2024). This discovery opens new avenues for understanding viral evolution and pathogenesis, and may lead to novel antiviral strategies targeting these interactions.

### 8.2 Multi-omics integration and systems biology

With only a fraction of miRNAs functionally characterized in farm animals, there is an urgent need for more focused research using advanced technologies (Ojo and Kreuzer-Redmer, 2023). Emerging approaches include:

• **CRISPR-Cas9 screening**: For functional characterization of miRNA targets
• **Single-cell sequencing**: For understanding cell type-specific miRNA functions
• **Integrated multi-omics approaches**: Combining miRNA profiling with genomics, transcriptomics, proteomics, and metabolomics (Ojo and Kreuzer-Redmer, 2023)

These technologies offer the potential to identify specific miRNA targets and develop novel pathways for early disease detection and mitigation strategies (Ojo and Kreuzer-Redmer, 2023).

### 8.3 Cross-disease convergence: a conceptual synthesis and priorities for future bioinformatic analysis

The disease-specific sections above point to a recurring pattern worth highlighting explicitly: a small number of miRNAs and their viral orthologs recur across otherwise unrelated veterinary viral diseases (Table 3). miR-155 and its viral ortholog mdv1-miR-M4-5p are repeatedly implicated in avian oncogenic transformation across MDV, ALV, and IBDV, consistent with a convergent oncogenic pathway across avian viral neoplasias (Bondada et al., 2019). Likewise, miR-21 has been independently linked to cell-survival signaling through PDCD4 repression in both MDV-induced lymphomagenesis (Stik et al., 2013) and PRRSV infection (Guo and Feng, 2025), raising the possibility of a shared regulatory node across otherwise unrelated viral families.

**Table 3:** Summary of selected veterinary viral diseases and associated miRNAs.

**Disease**	**Causative Virus**	**Host Species**	**Key miRNAs Involved**	**Functional Role of miRNAs**	**References**
PRRS	PRRSV (Arterivirus)	Swine	38 miRNAs identified: 27 antiviral (miR-181, miR-130b, miR-2911, miR-150, miR-320, miR-378, let-7 family) and 11 proviral (miR-296-3p, miR-373, miR-30c, miR-24-3p, miR-22)	Antiviral miRNAs suppress replication via viral genome targeting and IFN-I/NF-κB modulation; proviral miRNAs promote replication	Zhang et al., 2026; Guo and Feng, 2025
Foot-and-Mouth Disease	FMDV (Aphthovirus, Picornaviridae)	Cattle, swine, sheep, goats	bta-miR-17-5p, bta-miR-31, miR-1281	Modulate host antiviral response; indicate infection stage; potential antiviral targets	Stenfeldt et al., 2017; Dong et al., 2017
Bovine Viral Diarrhea	BVDV (Pestivirus, Flaviviridae)	Cattle	bta-miR-29b, bta-miR-2411, bta-miR-2904	Inhibit viral replication; regulate apoptosis and autophagy pathways	Fu et al., 2017
Marek's Disease	MDV (Gallid herpesvirus 2, Herpesviridae)	Chicken	gga-miR-21, miR-26a, miR-103-3p, miR-219b, mdv1-miR-M4-5p (viral ortholog of miR-155)	Tumorigenesis; immune modulation; apoptosis regulation; v-miRNA ortholog indispensable for tumor induction	Stik et al., 2013; Li et al., 2014a; Han et al., 2016; Zhao et al., 2017; Fotouh et al., 2026
Avian Leukosis	ALV (Alpharetrovirus, Retroviridae)	Chicken	gga-miR-221, gga-miR-222, gga-miR-375, miR-23, miR-34b-5p, miR-155 (activated by insertional activation)	Promote or suppress viral replication; regulate oncogenic pathways through insertional mutagenesis and NF-κB activation	Li et al., 2012; Li et al., 2014b; Dai et al., 2015; Li et al., 2017; Bondada et al., 2019
Infectious Bursal Disease	IBDV (Avibirnavirus, Birnaviridae)	Chicken	gga-miR-9*, gga-miR-2127-3p, gga-miR-130b-3p	Modulate viral replication; type I IFN production; host antiviral response	Ouyang et al., 2015; Ouyang et al., 2017; Fu et al., 2017
Ebola Virus Disease	Ebola virus (Filoviridae)	Humans, primates	miR-vp-3p (viral), hsa-miR-146a-5p, hsa-miR-21-3p, hsa-miR-29a-3p	Early detection via viral miRNA-like fragment before genomic RNA appears; host miRNAs as classifiers (86% accuracy)	Chen et al., 2016; Duy et al., 2016
Hendra Virus	HeV (Henipavirus, Paramyxoviridae)	Horses	miR-16, miR-32	Potential early diagnostic biomarkers; expression ratios improve discrimination	Cowled et al., 2017
Rabies	Rabies virus (Lyssavirus, Rhabdoviridae)	Dogs, Mice	miR-133, miR-200 family	Modulate viral latency in muscle and CNS; influence immune pathways	Zhao et al., 2012; Samir and Vaas, 2016
Newcastle Disease	OAVJ (Paramyxoviridae)	Poultry	vSMs, vSSs (viral)	Virulent strains encode more viral seed mimics and fewer seed sponges, correlating with pathogenicity	Mears and Bakre, 2024

The convergence pattern described above is proposed here explicitly as a working hypothesis rather than an established finding: the evidence reviewed is consistent with convergence but does not constitute a systematic, bias-controlled demonstration of it. Confirming or refuting this hypothesis would require a dedicated bioinformatic study: systematically compiling the differentially expressed miRNAs reported across the diseases covered in this review, followed by target prediction (e.g., TargetScan, miRDB), pathway enrichment analysis (e.g., DIANA-miRPath, KEGG), and protein–protein interaction network analysis (e.g., STRING), together with full reporting of the input miRNA list, software versions, statistical thresholds and multiple-testing correction, and public deposition of the underlying data and code. Such an analysis lies beyond the descriptive scope of the present narrative review, but represents a high-value and currently unmet priority for the field, and would substantially strengthen the evidence base linking specific miRNAs to shared host signaling pathways—such as type I interferon, NF-κB, and apoptosis signaling—across veterinary viral diseases.

#### 8.3.1 Recommendations for future bioinformatics priorities

In parallel with the disease-specific validation studies discussed in Section 6.5, we recommend three priority areas for the field: (1) development of species-specific miRNA target-prediction algorithms trained on experimental datasets from livestock, rather than relying solely on human-centric tools; (2) creation of an open-access veterinary miRNA-disease database to facilitate cross-disease meta-analyses of the kind outlined above; and (3) integration of miRNA profiles with transcriptomic, proteomic, and metabolomic data using multi-omics approaches to capture the full complexity of host–virus regulatory networks (Ojo and Kreuzer-Redmer, 2023).

### 8.4 One health perspectives: bridging human and veterinary miRNA research

The One Health framework recognizes the interconnectedness of human, animal, and environmental health–a perspective particularly relevant to miRNA research, given the high sequence conservation of miRNAs across mammalian and avian species (Samir and Pessler, 2016). Rather than treating human and veterinary research as parallel tracks, we propose a **"**One miRNA, One Health" model based on shared molecular mechanisms and interchangeable biomarkers. This section introduces this convergence model as a novel framework that moves beyond simple sequence conservation to propose a concrete, three-phase roadmap for cross-species translation of miRNA-based diagnostics and therapeutics (see Section 8.4.4).

#### 8.4.1 Conserved miRNA signatures in zoonotic diseases

Three zoonotic viruses—Ebola virus, Hendra virus, and influenza A virus (IAV)—provide compelling examples of conserved miRNA responses across species:

• **Ebola Virus:** The host miRNA response (miR-146a-5p, miR-21-3p, miR-29a-3p) shows substantial overlap between human patients and macaque models (Duy et al., 2016). This conservation suggests potential for cross-species biomarker development, though a panel validated in humans would still require independent confirmation in non-human primates before diagnostic use.
• **Influenza A Virus:** miR-144 and miR-451 are consistently upregulated in both swine and human IAV infections, targeting the viral NP gene and inhibiting replication (Dong et al., 2017). This shared mechanism suggests that miRNA-based antivirals could have dual human-veterinary applications, potentially accelerating regulatory approval pathways.
• **Hendra Virus:** miR-16 and miR-32 were validated as candidate biomarkers in experimentally infected horses (Cowled et al., 2017). Because bats are the natural reservoir of HeV, testing whether homologous miRNA responses occur in bats represents a promising, currently untested opportunity for eco-surveillance of this and other bat-borne pathogens.

#### 8.4.2 Practical one health applications

**(a) Early Warning Systems for Zoonotic Spillover:** Monitoring miRNA biomarkers in sentinel animals (e.g., horses for HeV, pigs for IAV, bats for Ebola) could in principle allow detection of enzootic circulation of zoonotic viruses before human cases emerge, building on experimental evidence that circulating miRNA changes are detectable during HeV infection in horses (Cowled et al., 2017). Prospective field studies are needed to determine whether such a sentinel-surveillance approach can detect subclinical infection with adequate lead time under real-world conditions before it could be recommended for routine use.

**(b) Cross-Species Therapeutic Development:** The high sequence identity of many miRNAs raises the possibility that insights from therapeutic development in one species could inform work in others, although this remains largely a conceptual opportunity rather than an established practice. The clearest existing precedent for silencing a conserved host miRNA as an antiviral strategy is the anti-miR-122 compound miravirsen, whose antiviral efficacy against hepatitis C was first established in HCV-infected chimpanzees before progressing to human trials (Lanford et al., 2010). No comparable veterinary application of this strategy has yet been reported, and any such effort would require dedicated species-specific safety and efficacy studies rather than direct extrapolation from the human/primate experience.

**(c) Integrated Surveillance Platforms:** Given the conservation of miRNAs across species, veterinary diagnostic laboratories can serve as sentinel sites for human public health. A veterinary laboratory that routinely assays samples for livestock diseases can be equipped to detect miRNA signatures of zoonotic viruses in the same samples, creating an integrated surveillance network without redundant infrastructure investments (Table 4).

**Table 4:** Applications of miRNAs in veterinary viral infections.

**Application**	**miRNA-Based Strategy**	**Mechanism / Role**	**Example from Study**	**References**
Diagnostic biomarker	Circulating miRNA profiling	Reflects infection stage, subclinical carrier status	bta-miR-17-5p and bta-miR-31 in FMDV-infected cattle; miR-16 and miR-32 in HeV	Stenfeldt et al., 2017; Cowled et al., 2017; Dong et al., 2017
Diagnostic biomarker	Urinary exosomal miRNA	Non-invasive liquid biopsy for companion animal diseases	Urinary miRNAs for CKD in dogs and cats	Chu and Nabity, 2025
Antiviral therapy	miRNA inhibition	Suppresses disease-promoting miRNAs	Antagomirs and LNA-based inhibitors targeting oncomiRs or virus-favoring miRNAs	Lima et al., 2018; Stenvang and Kauppinen, 2008
Antiviral therapy	miRNA replacement	Restores tumor suppressor or antiviral miRNAs	miR-29b replacement inhibits BVDV replication	
Antiviral therapy	Viral seed mimic/sponge targeting	Disrupts viral hijacking of host miRNA pathways	Targeting vSS-miR-27b-5p alters viral gene expression	Mears and Bakre, 2024
Vaccine development	miRNA-based adjuvant	Modulates immune signaling pathways, enhances antigen response	Synthetic miRNAs co-delivered with antigens	Mochida and Uchida, 2024
Vaccine development	miRNA-regulated gene delivery	Restricts transgene expression to target tissues	Effective in poliovirus, influenza A, flaviviruses, dengue virus	Dhungel et al., 2018
Early infection detection	Viral miRNA or miRNA-like fragments	Detects viral activity before genomic RNA is detectable	miR-vp-3p in Ebola virus infection	Chen et al., 2016
Host-pathogen interaction studies	Profiling host and viral miRNAs	Identifies regulatory pathways during viral replication	miR-9* enhances IBDV replication by suppressing IFN production	Ouyang et al., 2015
Genetic selection for disease resistance	miRNA-targeted breeding	Identifies miRNA polymorphisms associated with disease resistance	Potential for improving chicken disease resistance through miRNA regulation	Fotouh et al., 2026

#### 8.4.3 Challenges and priorities for one health translation

Despite conceptual appeal, practical implementation of One Health miRNA approaches faces substantial barriers:

1. **Regulatory fragmentation:** Human and veterinary medical regulatory agencies operate independently; no framework exists for dual approval of miRNA-based diagnostics or therapeutics for both human and animal use.
2. **Financial incentives:** Human diagnostics are typically reimbursed at higher rates than veterinary equivalents, creating a "valley of death" where promising veterinary biomarkers are not commercialized due to insufficient market incentives.
3. **Species-specific environmental factors:** Nutrition, housing, and microbiome composition differ between humans and animals, potentially altering miRNA stability and function across species.
4. **Ethical considerations:** The use of sentinel animals for human disease surveillance raises ethical questions about animal welfare and informed consent.

#### 8.4.4 A Roadmap for one health miRNA integration

To address these challenges, we propose a three-phase roadmap:

• **Phase 1 (2026-2028):** Establish a global consortium for veterinary miRNA research (GVMR) to harmonize protocols, standardize reference databases, and coordinate multi-species validation studies.
• **Phase 2 (2028-2031):** Develop and validate miRNA panels for zoonotic viruses in at least three model species (e.g., swine, poultry, horses) with simultaneous human data collection to establish cross-species equivalence.
• **Phase 3 (2031-2035):** Advocate for joint human-veterinary regulatory pathways and the inclusion of miRNA-based surveillance in international health regulations.

### 8.5 Practical considerations for field application

Despite the excitement about utilizing non-coding RNAs to combat animal viral diseases, considerable challenges need to be overcome before clinical use (Kappari et al., 2024):

• **Delivery to large groups:** Most veterinarians prefer antiviral therapies delivered through common-source bio-vehicles (e.g., food, water, or air) for quick accessibility. Individual miRNA-based therapies are more practical for high-value animals (e.g., racehorses, SPF parent flocks) (Kappari et al., 2024)
• **Speed of action:** RNAi produces inhibitory effects within 1–2 days in cell lines, which may be insufficient for rapidly spreading diseases like FMDV and IAV (Kappari et al., 2024)
• **Toxicity concerns:** Excessive miRNA levels can saturate endogenous processing machinery, giving rise to toxicity, pathology, and mortality (Kappari et al., 2024)
• **Viral escape:** Rapidly mutating RNA viruses may escape miRNA-mediated suppression, necessitating the use of pooled siRNAs or cocktails targeting both viral and host genes (Kappari et al., 2024)

## 9 Limitations and challenges

### 9.1 Reference data limitations

A major limitation is the scarcity of high-quality reference datasets for livestock species. Compared with humans, many domestic animal species still lack well-annotated reference genomes and comprehensive miRNA databases (Ojo and Kreuzer-Redmer, 2023). While 962 miRNAs have been annotated for dogs in miRBase, no miRNAs from cats have been annotated, highlighting a significant gap in companion animal miRNA research (Ojo and Kreuzer-Redmer, 2023). This limitation complicates miRNA identification, annotation, and functional characterization while reducing the accuracy of target gene prediction (Samir and Pessler, 2016; Samir and Vaas, 2016).

### 9.2 Biological and pre-analytical variability

miRNA expression profiles can be influenced by multiple factors, including age, sex, breed, physiological status, and environmental conditions (Do et al., 2021). Such variability reduces reproducibility across studies and complicates the identification of reliable species-specific biomarkers (Do et al., 2021). Interspecies differences in host responses to viral infection limit the direct translation of findings across animal species (Dong et al., 2017).

**Pre-analytical factors** that significantly affect miRNA quantification include:

• Sample collection methods and anticoagulant selection (McDonald et al., 2011)
• RNA extraction methods (Guo et al., 2017)
• Detection platforms and normalization strategies (Blondal et al., 2013)
• Storage conditions and freeze-thaw cycles (Glinge et al., 2017)
• Hemolysis and sample quality (Pritchard et al., 2012)

### 9.3 Technical and analytical challenges

The small size and low abundance of miRNAs necessitate highly sensitive and standardized analytical approaches for accurate quantification (Ho et al., 2022). Technical variability in sample pooling, sequencing depth, multiplexing, and experimental workflow can significantly impact results (Ho et al., 2022). The presence of isomiRs adds another layer of complexity to miRNA detection and quantification (Tomasello et al., 2021).

### 9.4 Therapeutic translation barriers

The use of synthetic miRNAs, miRNA mimics, and antagomirs requires overcoming multiple barriers:

• Nuclease-mediated degradation and rapid systemic clearance (Eberle et al., 2008; Hornung et al., 2005)
• Off-target effects and passenger-strand loading (Søkilde et al., 2015)
• Inefficient tissue-specific delivery
• Functional complexity: a single miRNA may regulate multiple genes and signaling pathways simultaneously (Do et al., 2021)
• Saturation of endogenous miRNA processing machinery (Kappari et al., 2024)

### 9.5 Economic and practical constraints

The practical implementation of miRNA-based therapies in production animal settings faces unique economic and logistical challenges (Kappari et al., 2024). Most commercial animal production systems are designed for mass administration of therapeutics through feed or water, not individual dosing. The cost of synthetic miRNA molecules and specialized delivery systems may be prohibitive for routine use in food animal production (Kappari et al., 2024).

### 9.6 Integrated SWOT analysis

The application of miRNA-based approaches in veterinary virology presents a promising yet challenging frontier, as highlighted by the SWOT analysis. Internally, these methods offer significant strengths, including the high stability of miRNAs in serum and plasma, cross-species conservation that facilitates broad application, and the potential for minimally invasive liquid biopsies, alongside established chemical modification techniques such as LNA and 2′-OMe. However, these advantages are counterbalanced by critical weaknesses, including the absence of validated veterinary-specific miRNA databases, considerable inter-study heterogeneity, a low technology readiness level (TRL ≤4) for all current candidates, and practical hurdles such as the logistical impracticality of mass delivery and the risk of TLR7/8 immunostimulation. Externally, there are substantial opportunities to leverage advanced tools like CRISPR screens for target discovery, reuse existing data from public repositories such as GEO and miRBase, capitalize on decreasing NGS costs for broad screening, and utilize AI/ML for panel optimization, all while aligning with the One Health initiative for cross-species translation. Nevertheless, these opportunities are threatened by the high mutation rate of viruses, which could engender resistance, regulatory uncertainty surrounding RNA therapeutics in food-producing animals, public concerns over consumer residues, and stiff competition from cheaper, conventional diagnostic methods.

## 10 Conclusion and future perspectives

The evidence synthesized in this review highlights the expanding translational potential of microRNAs in veterinary viral diseases. Circulating miRNAs have emerged as promising biomarkers due to their stability, accessibility, and ability to reflect disease-associated molecular changes, supporting their application in early diagnosis, disease monitoring, and therapeutic evaluation (Dong et al., 2017; De Guire et al., 2013). At the same time, miRNA-targeted strategies offer innovative opportunities to modulate host regulatory pathways and complement existing antiviral approaches (Zhang et al., 2026; Samir and Pessler, 2016).

**Key Findings:**

1. miRNAs exhibit dual roles in viral infection—functioning as either antiviral or proviral factors depending on context, cell type, and infection stage (Zhang et al., 2026; Guo and Feng, 2025)
2. Virus-encoded miRNAs and viral seed mimics/sponges represent sophisticated viral strategies to manipulate host regulatory networks (Fotouh et al., 2026; Mears and Bakre, 2024)
3. The remarkable stability and cross-species conservation of many miRNAs support their development as biomarkers across veterinary species (Samir and Pessler, 2016; Hall et al., 2012)
4. miRNA-based therapeutic strategies, including inhibition, replacement, and regulated gene delivery, show promise in preclinical studies (Bernardo et al., 2015; Dhungel et al., 2018)
5. Integrative bioinformatics analysis reveals that despite disease-specific variations, miRNAs converge on a limited set of core signaling pathways governing innate immunity, inflammation, and cell survival

**Critical Gaps Requiring Urgent Attention:**

1. Development of veterinary-specific reference databases and comprehensive miRNA annotations for domestic species (Ojo and Kreuzer-Redmer, 2023)
2. Standardization of analytical protocols for miRNA detection, quantification, and normalization (Blondal et al., 2013)
3. Large-scale multicenter validation studies to confirm candidate biomarkers (Do et al., 2021)
4. Advancement of safe, efficient, and cost-effective delivery systems suitable for field application (Kappari et al., 2024)
5. Establishment of regulatory pathways for joint human-veterinary miRNA-based diagnostic and therapeutic products

**Future Directions:**

1. **Multi-omics integration**: Combining miRNA profiling with genomics, transcriptomics, and proteomics using advanced technologies like CRISPR-Cas9 screening, single-cell sequencing, and integrated multi-omics approaches (Ojo and Kreuzer-Redmer, 2023)
2. **Artificial intelligence**: Machine learning approaches for miRNA target prediction, biomarker discovery, and patient stratification
3. **One Health applications**: Given the conservation of miRNAs across species, miRNA-based approaches could simultaneously benefit human and animal health—particularly for zoonotic diseases (Dong et al., 2017)
4. **Rapidly acting RNAi technologies**: Development of more rapidly acting RNAi to control highly contagious infections like FMD (Kappari et al., 2024)
5. **Practical delivery systems**: Incorporation of RNAi molecules into animal feed or water supply for mass administration (Kappari et al., 2024)

**Short-term actionable priorities (2025–2028):**

(1) Publish a consensus pre-analytical protocol for circulating miRNA quantification in livestock biofluids to overcome the heterogeneity identified in Section 5.3.
(2) Build an open-access veterinary miRNA disease database with standardised metadata (as per Section 8.3.1) to facilitate the cross-disease meta-analyses proposed here.
(3) Conduct CRISPR-based functional screens in species-relevant cell systems (Sanjana et al., 2014) to define the targetomes of miRNAs such as miR-155 and miR-146a in specific veterinary cell types.
(4) Establish multi-species natural-infection validation cohorts (≥100 animals per disease-status group) to progress the candidate biomarkers in Table 1 from the discovery phase (TRL 1-3) to preclinical validation (TRL 4-6).
(5) Engage with EMA/USDA-APHIS to define a clear regulatory pathway for RNA veterinary therapeutics, including residue/withdrawal considerations for food animals.

**Prospects for Clinical Translation:**

While miRNA-based technologies are well positioned to become important components of next-generation veterinary diagnostics and therapeutics, substantial translational barriers remain. The search for target sequences conserved across viral strains—particularly for rapidly mutating RNA viruses—is of highest priority. Using pools of various siRNAs or cocktails specific for both viral and host genes may reduce escape of mutant viruses (Kappari et al., 2024). As experimental evidence continues to accumulate and technical challenges are progressively addressed, miRNA-based approaches hold significant promise for advancing precision veterinary medicine and improving disease surveillance, prevention, and control within the One Health framework.

Continued integration of miRNA research with genomics, bioinformatics, artificial intelligence, and multi-omics technologies is expected to accelerate progress. Within the One Health paradigm, these advances may improve disease surveillance, support more effective prevention and control strategies, enhance animal health and welfare, strengthen food security, and contribute to global public health.

## Funding

No external funding was received for this study. All research-related expenses, including publication costs, were fully covered by the authors.

## Declaration of generative AI and AI-assisted technologies in the writing process

During the preparation of this work, the authors used Claude AI (Anthropic) for language editing, structural organization, and formatting. Following the use of this tool, the authors reviewed and revised the content as necessary and take full responsibility for the final submitted manuscript.

## CRediT authorship contribution statement

**Mohamad Reza Choobineh:** Writing – original draft, Visualization, Resources, Methodology, Investigation, Data curation. **Daryoosh Choobineh:** Writing – original draft, Visualization, Resources, Investigation. **Behzad Hajieghrari:** Writing – review & editing, Writing – original draft, Supervision, Conceptualization.

## Declaration of competing interest

The authors declare that they have no known competing financial interests or personal relationships that could have appeared to influence the work reported in this paper.

## Data Availability

Data will be made available on request.
